# High contextual interference improves retention in motor learning: systematic review and meta-analysis

**DOI:** 10.1038/s41598-024-65753-3

**Published:** 2024-07-10

**Authors:** Stanisław H. Czyż, Aleksandra M. Wójcik, Petra Solarská, Paweł Kiper

**Affiliations:** 1 Faculty of Physical Education and Sports, Wrocław University of Health and Sport Sciences, Wrocław, Poland; 2 Faculty of Sport Studies, Masaryk University, Brno, Czechia; 3 Physical Activity, Sport and Recreation (PhASRec), North-West University, Potchefstroom, South Africa; 4 Healthcare Innovation Technology Lab, IRCCS San Camillo Hospital, Venezia, Italy

**Keywords:** Psychology, Human behaviour

## Abstract

The effect of practice schedule on retention and transfer has been studied since the first publication on contextual interference (CI) in 1966. However, strongly advocated by scientists and practitioners, the CI effect also aroused some doubts. Therefore, our objective was to review the existing literature on CI and to determine how it affects retention in motor learning. We found 1255 articles in the following databases: Scopus, EBSCO, Web of Science, PsycINFO, ScienceDirect, supplemented by the Google Scholar search engine. We screened full texts of 294 studies, of which 54 were included in the meta-analysis. In the meta-analyses, two different models were applied, i.e., a three-level mixed model and random-effects model with averaged effect sizes from single studies. According to both analyses, high CI has a medium beneficial effect on the whole population. These effects were statistically significant. We found that the random practice schedule in laboratory settings effectively improved motor skills retention. On the contrary, in the applied setting, the beneficial effect of random practice on the retention was almost negligible. The random schedule was more beneficial for retention in older adults (large effect size) and in adults (medium effect size). In young participants, the pooled effect size was negligible and statically insignificant.

## Introduction

One of the variable practice phenomena is that more variability initially hinders learning but, in many cases, subsequently benefits it^1^. Training schedule is one of the sources of variability^1^. One of the effects of variability caused by the training schedule is called the contextual interference (CI) effect^1^.

The CI effect has been exhaustively studied since the original Battig’s publication in 1966^2^. Battig reported that immediate performance, retention, and transfer were affected by practice organizations differently, depending on whether they were scheduled in random or blocked order. When the practice was organized in so-called “*random order*”, it consisted of the training trials arranged randomly with rapidly changing order. It was defined as a high CI. The term *random practice* is interchangeably used with *interleaved practice*^3,4^. On the other hand, the term blocked order was used when trials corresponding to one skill variation were completed before introducing the next skill. The blocked order, sometimes called *repetitive practice*^5^, was defined as a low CI.

Battig^2^ noted that random practice condition hinders performance during acquisition, although it facilitates retention and transfer. Participants learning in high CI conditions had better retention and transfer results than the low CI practice groups. Battig focused on verbal learning^2^; however, specialists in motor learning realized the importance of these findings. The first study using a motor task was conducted by Shea and Morgan^6^. In their study, now considered classical in motor learning, participants learned three motor barrier knock-down tasks (the purpose of the task was knocking down a specified number of six freely moveable barriers in a prescribed order) in a blocked (low CI) or random (high CI) order. The reaction and movement time (and total time) were measured, evincing that, during acquisition, these times were shorter in the low CI group. Conversely, in retention tests, these times were the longest for the low CI group, tested in a random order, i.e., tested in high CI^6^. Such discovery, supported by tens of similar findings, had a profound effect in laboratory and field-based studies. Many motor learning books suggest that implementing high CI in practice may enhance learning and transfer^7–10^. Despite the fact that there were studies questioning the positive effect of the high CI on retention and transfer or at least showing its relatively small effect size in field studies^7,11^, most of the reviews and publications focusing on the CI effect in motor learning suggest that this effect is robust across tasks and fields^12–16^ marginally mentioning the limitations of the CI effect (compared to the emphasized benefits).

Recently, the CI effect aroused interest among researchers focusing on the neurophysiology of motor learning. Their studies reported differences in Central Nervous System (CNS) regions activations while acquiring skills under blocked and random conditions^17^. These findings sparked an interest in contextual interference topic among more extensive group of researchers.

In 2004, Brady^7^ formulated first doubts about the CI effect. Considering ES in basic research (Cohen’s d = 0.57) and applied research (Cohen’s d = 0.19), Brady noted that future studies should answer the question asked by Al-Mustafa^11^ whether CI is a laboratory artifact or sport-skill related.

In 2023, Ammar and colleagues published their meta-analysis^18^. Unfortunately, it was poorly performed. For example, they searched the Taylor and Francis database, though it is a publisher base, not a scientific one. At the same time, they did not screen the EBSCO database, which consists, among the others, of APA PsycArticles, APA PsychInfo, SPORTDiscus with Full Text, Medline, and Academic Search Complete. Their review was not preregistered, which is a standard procedure these days. Given these methodological flaws, the review of Ammar et al. cannot be considered reliable and valid.

Many of the narrative reviews^7,12–16,19,20^ did not specify what search terms were used to identify the included studies. The authors did not report the effect sizes of the reviewed studies, either. In a meta-analytic review on CI by Lage^21^, the authors presented all of the aforementioned components: search terms were specified, and effect sizes of the studies have been reported. Nevertheless, no *blocked* practice schedule was included, as the study's primary goal was to compare the effect of *random* and *serial* practice on transfer and retention.

The meta-analyses on CI conducted by Graser^22^ and Sattelmayer^23^ provided the inclusion criteria. However, population criteria were restricted to children with cerebral palsy^22^ or students in medical and physiotherapy education^23^.

Given all of the above, it is crucial to update and perform a meta-analysis focusing on the CI effect on retention in general population since retention was the most conspicuous and paradoxical effect reported by Battig^2^. We hope this meta-analysis may help solve some of the controversies about the CI effect in motor learning. 

Therefore, the study's primary objective is to determine the effect of CI on retention in motor learning, as it was initially the main objective of Shea and Morgan study^6^. In order to be consistent with previous Brady’s meta-analysis^7^, we formulated secondary objectives based on Brady’s inclusion criteria, i.e.:

1. To estimate the CI effect in laboratory vs. field-based studies
2. To estimate the CI effect in young vs. adults vs. elderly adults
3. To estimate the CI effect in novice vs. experienced participants

## Methods

The study was registered in PROSPERO under the number CRD42021228267. The review was conducted according to PICO guidelines^24^, the PRISMA Statement^25^ and was supplemented by the *Quality Assessment Tool for Quantitative Studies*^26^.

Due to number of included studies, the review has been split into two consecutive papers: retention and transfer meta-analyses. Retention performance was analyzed in the present study.

### Inclusion and exclusion criteria

Eligible studies were identified based on PICO (*Population*, *Intervention*, *Control*, *Outcome*):

Population: young, adult, novice, experienced. Only healthy participants, as classified by authors of the primary studies, were included. We did not include studies on disabled participants. The population criteria were divided into two variables. The first variable was related to the age of the participant. Participants classified as *Young* were under 18 years old, those between 18 and 60 years old were classified as *Adults*. *Older Adults* were participants over 60 years old. The second variable was experience. We recognized *Novice* and *Skilled* (*Experienced*) participants according to the authors’ statement.

Intervention: high CI (random/interleaved schedule); field setting.

Control: low CI (blocked schedule/ repetitive practice); laboratory settings.

The studies utilized a wide variety of motor tasks and experimental procedures. However, only the single-task procedure (as opposed to dual-task procedure, only one task is performed at a time) was considered relevant for this review.

The main category considered for analysis was the contextual interference—studies including groups with different practice order: random schedule (high CI) and blocked schedule (low CI) were compared.

The subgroup analysis was performed. Firstly, the intervention category was related to the nature of research—studies conducted in a field setting using typical sports skills (applied research) were matched up with studies carried out in a controlled laboratory environment (basic research). The second category of subgroup analysis was the age of the participants: young (< 18 y), adults and older Adults (≥ 60 y). The third category was the experience—experienced participants vs. novices.

Outcome: retention test results. The primary outcomes were the standardized effect sizes of CI in retention in motor learning. The outcomes evaluating retention of the learned motor skill were considered selectable. Considering the effect of *sleep-induced consolidation of trained skills*^27,28^, meta-analysis consisted of delayed retention results only, i.e., results of the tests performed after 24 h while systematic review consisted of immediate and delayed retention test results. We assumed that most of the analyzed study participants should have slept between acquisition and retention tests during the last 24 h. As Diekelmann and Born^27^(p.114) noticed, “*Sleep has been identified as a state that optimizes the consolidation of newly acquired information in memory, depending on the specific conditions of learning and the timing of sleep.*”, hence the CI effect should more conspicuous after memory consolidation. Studies describing immediate retention results were discussed in the systematic review.

### Search methods and selection procedure

AW performed the search on the following databases: Scopus (“contextual interference” in Title OR Abstract OR Keywords), EBSCO (“contextual interference” in Title OR Abstract—no Keywords option), Web of Science (“contextual interference” in Topic), ScienceDirect (“contextual interference” in Title OR Abstract OR Keywords), in April 2020 (for the period 1966 to 2020), updated in September 2021 (period 2020 to 2021) and November 2022 (period 2021–2022). Additionally, relevant studies were scrutinized using the Google Scholar search engine (“contextual interference” in Title OR Abstract OR Keywords). PsycINFO (EBSCO) database was searched by SC (“contextual interference” in Title OR Abstract OR Keywords).

For the reliable risk-of-bias assessment, the “grey literature” (i.e., Ph.D. dissertations and conference proceedings) available on-line in the searched databases has been excluded as well as studies in languages other than English.

Given the large number of retrieved citations we applied a method proposed by Dundar and Fleeman^29^. AW evaluated all the titles, keywords, and abstracts of the studies for inclusion and exclusion criteria and a random sample was cross-checked by senior researcher (SC). Ineligible articles were excluded. Duplicates of identified studies have been removed.

Two co-authors (AW and PS) read the full text of the studies, independently assessing the papers for final eligibility. Any discrepancies between the two authors were discussed with the senior researcher (SC) to reach a consensus.

### Data collection and analysis

AW and PS summarized relevant data in developed MS Excel data extraction forms during the screening. Each entry consisted of study characteristics: the authors' names, the title of the study, year of publishing, journal title, and number of experiments (in case of multiple experiments in the same publication). Further details were based on PICO criteria:

- *Population* (number of participants, age, expertise level, gender),

- *Intervention* (nature of research, practice schedule, type of motor task, testing procedure, dependent variable),

- *Objectives/outcomes* (extracted means and standard deviations for all groups and all measures -immediate retention results and delayed retention results). Only the results of the first block in the retention testing procedure were considered for extraction. We assumed that the following blocks may promote further learning. If SEM (standard error of the mean) was available, we converted it into SD. Similarly, if quartiles were available, we converted these with Mean Variance calculator^30–33^. Whenever required, the positive/negative effect sizes were transformed to ensure that positive always favors random practice.

We included the results from both, blocked and random scheduled retention tests. If participants were tested in both conditions, both results were included independently. In addition, based on the *Quality Assessment Tool for Quantitative Studies* (described in the following section), study quality indicators were included (covering: selection bias, study design, confounders, blinding, data collection methods, withdrawals, and dropouts, global rating).

Since the included studies utilized different motor skills (tasks), retention was measured using different scores (numbers, percentages) or units (seconds, meters, number of cycles, etc.), we summarized the analysis using standardized mean difference (SMD) as an effect size measure, i.e. Hedges’ (adjusted) g, very similar to Cohen's d, but it includes an adjustment for small sample bias^34,35^.

Heterogeneity among the studies was evaluated using *I*^*2*^ statistics. The interpretation of I^2^, is as follow: 30% to 60% represent moderate heterogeneity; 50% to 90%—substantial heterogeneity; and 75% to 100%—considerable heterogeneity^36^. However, thresholds for interpretation can be misleading^37^.

Following guidelines were applied while interpreting the magnitude of the SMD in the social sciences: small, SMD = 0.2; medium, SMD = 0.5; and large, SMD = 0.8^38^.

Given that the studies included in our analyses could yield more than one outcome, we decided to use a model that accounts for various sources of dependence, such as within-study effects and correlations between outcomes. Since there is no universally accepted method for this, we opted to apply two different approaches, each with its own advantages and disadvantages. The first approach is based on a three-level mixed model. The second approach is a classical random-effects model based on averaged outcomes (SMDs) from a single study.

#### Three-level mixed model

A three-level mixed model which uses (restricted) maximum likelihood procedures^39,40^ was computed. The model considers the potential dependence among the effect sizes, i.e. when there are multiple outcomes (effect sizes) from one study. The model assumes that the random effects at different levels and the sampling error are independent. The first level of the model refers to variance between effect sizes among participants (level 1). The second level refers to outcomes, i.e. effect sizes extracted from the same study (level 2; within-cluster variance). The third level refers to studies (level 3; between-clusters variance)^39^. Given the second level in the model accounts for sampling covariation, the benefit of this model is that it is no crucial to know or estimate correlations between outcomes from extracted one study^39,41^.

Sensitivity analysis was performed using Cook’s D distances. Outcomes further than 4/n (where n was the number of outcomes) were removed to assess how these outliers influence the pooled effect. Meta-analyses were performed with RStudio (version 2023.06.0) and the following packages “metaphor”, “dmetar”, “tidyverse”, “readxl”, and “ggplot”.

#### Random-effects model with averaged outcomes (effect sizes)

We applied a random effects model which estimates the mean of this distribution of true effect sizes. We applied the model based on averaged outcomes, whenever these outcomes were drawn from the same population (same study). We computed average standard errors using variances and then took the square root, as this approach typically yields the most meaningful results. In this approach, the problem of potential correlations between results is resolved. However, the limitation of this method is that averaging effect sizes from a single study reduces the variances between them, and informative differences between outcomes are lost^42,43^.

Like in the previous analysis, sensitivity analysis was performed using Cook’s D distances. Meta-analyses were performed with RStudio (version 2023.06.0) and the following packages “tidyverse”, “readxl”, “metaphor”, “dmetar”, and “meta”.

### Assessment of risk of *bias*/quality assessment in included studies

The risk of bias in included studies has been assessed using the Effective Public Health Practice Project (EPHPP) *Quality Assessment Tool for Quantitative Studies*^44^. The checklist components are as follows: sample selection, study design, identification of confounders, blinding, reliability and validity of data collection methods, as well as withdrawals and dropouts. These elements can be rated strong, moderate, or weak, corresponding to a standardized guide and dictionary. The comprehensive evaluation of the study is determined by assessing six rating aspects. Studies with no weak ratings and at least four strong ratings are regarded as strong. Those with less than four strong ratings and one weak rating are considered moderate, and those with two or more weak ratings are considered weak^26^.

AW and PS independently assessed the level of evidence and methodological quality of the eligible studies (Appendix 1, https://osf.io/r59zs/?view_only=61397e4508384d13960936a556890962). In case of disagreement, the authors discussed until consensus was reached. If any hesitance arose regarding the quality of the study, the problem was discussed with the senior researcher (SC).

## Results

### Results of the search

**Figure 1:**
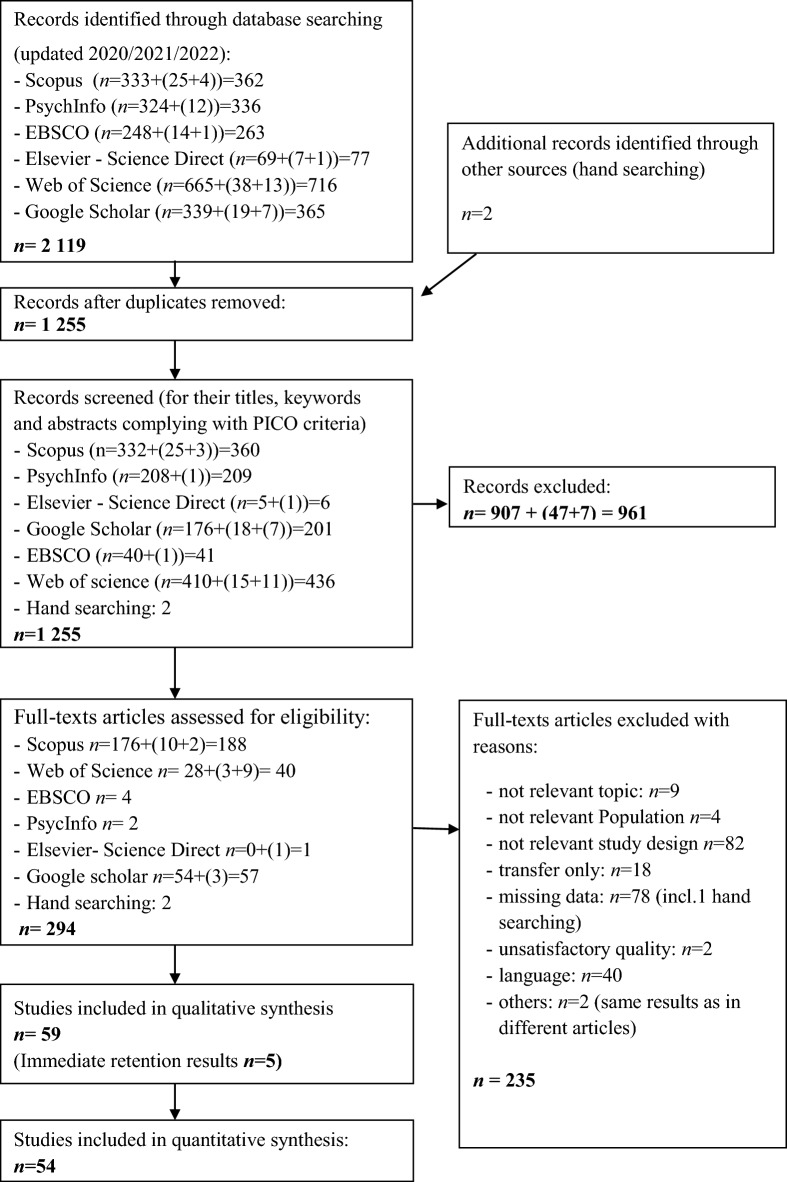
PRISMA flow diagram of the search process^45^. Flowchart of the primary search (time period 1966 to 2020), updated searches (time period 2020 to 2021 and 2021 to 2022), and the inclusion and exclusion process.

The primary search on electronic databases identified 2119 potential records. After removing duplicates, titles and abstracts of 1255 articles were screened according to PICO criteria, of which 961 records were excluded due to topic reasons, study design issues, and population. The detailed evaluation process is highlighted in the PRISMA Flow Diagram^45^ (Fig. 1).

The remaining 294 studies were evaluated, and 235 of these were excluded (detailed reasons for exclusion are listed in Appendix 2, https://osf.io/r59zs/?view_only=61397e4508384d13960936a556890962). Four studies did not match population criteria and were excluded (participants with hyperfunctional voice disorders, participants with Parkinson’s disease, children with Developmental Coordination Disorder, individuals with visual impairments). The topics of nine studies were not relevant to the main objectives of our review (related to agility training, chunking process, neural network simulation, the effect of cues, observational learning, feedback, awareness of maximal fluidity). Eighty-two articles were excluded because the study design was not relevant to PICO criteria. Two studies were excluded as the authors either did not provide details on dependent measures or did not inform how the dependent variables were quantified.

Seventy-eight studies were not included due to missing data. In case data were not found in the article, the authors were contacted via e-mail and/or the ResearchGate platform. Ninety-six data requests have been sent, and thirty-eight answers (about 39 articles) were received. Thirteen of theses replays did not report the data, mainly due to a long time since publication. However, data related to twenty-five articles were successfully obtained.

Eighteen articles did not cover retention testing, focusing mainly on transfer. In 40 studies, only abstracts were presented in English. The results of two articles were similar to the same authors’ other articles’ outcomes.

Finally, 59 studies were included in the present systematic review, however the quantitative analysis covered 54 articles. Retention tests up to 24 h after the last acquisition session were defined as *immediate retention* testing. Accordingly, tests performed after 24 h and more were categorized as *delayed*. Five studies described immediate retention testing. These results were not included in the meta-analysis. An overview of all included studies is provided in Table 1.

**Table 1:** Summary of the included studies.

	Authors	Number of experiment/ group characteristics	Testing procedure	Time interval between acquisition and testing	Measurement/dependent variable	Type of study	N	Objectives						
								Age	Students (yes/no)	Experience	Nature of research	Measure ratio	Type of motor task	p-value
1	Thomas, J.L., Fawver, B., Taylor, S., Miller M.W., Williams, A.M., Lohse, K.R, 2021	1/with error estimation		(24 h)	AE (absolute error)	Randomly assigned	84 in total	22.95 ± 3.24 (< 35)	Yes	Novice	Lab	Ms	Time estimation task	
		1/no error estimation						21.26 ± 2.38 (< 35)						
2	(A) Beik, M., Fazeli, D. 2021	Similar		(24 h)	Absolute constant error (the lower the better)	Randomly assigned	40 in total	67.68 ± 3.95 (60–75)	No	Novice	Lab	Ms	Sequential timing task	
		Dissimilar												
3	Immink, M.A., Pointon, M., Wright, D.L., Marino, F.E., 2021	1. Block		(24 h)	Mean response time (the lower the better)	Randomly allocated	26	22.9 ± 5,2	N/A	Novice	Lab	Ms	Sequence learning task	
4	Kim, T., Kim, H., Wright, D.L., 2021	1. "Sham" groups only	Repetitive	(6 h)	Total time (TT) (The lower the better)	Randomly assigned	36	18 > (undergraduate students)	Yes	Novice	Lab	Ms	Discrete sequence production task (DSP)	
				(24 h)										
5	Schorn, J.M., Knowlton, B.J. , 2021* 2 experiments—2nd experiment excluded due to transfer results and missing information (number of participants)	1	Blocked	(24 h)	Reaction time (RT)	Randomly assigned	83	20.6 ± 3.2 (18–43)	Yes	Novice	Lab	Scores	Serial reaction time task (SRTT)	
					Accuracy (the higher the better)									p = 0.968
			Interleaved	(24 h)	Accuracy									p = 0.234
6	(B) Beik M., Taheri H., Ghoshuni M., 2021	1/similar condition		Immediate	Absolute error (the lower the better)	Randomly assigned	40 in total	67.68 ± 3.95 (60–75)	No	Novice	Lab	Ms	Sequential motor task	
		1/dissimilar condition												
		1/similar condition		24 h										
		1/dissimilar condition												
		1/ Similar Condition		Immediate	Variable error (the lower the better)									
		1/dissimilar condition												
		1/similar condition		24 h										
		1/dissimilar condition												
7	Porter C., Greenwood D., Panchuk D., Pepping G.J., 2020	1	Random	Immediate	Percent of shooting success (the higher the better)	Randomly assigned	24	24.9 ± 3.3 (21–34)	No	Not representative level (1.1 ± 1.3 years of experience)	Applied	Shooting success (%)	Basketball set shot	
				(7 days)										
8	Chua L.K., Dimapilis M.K., Iwatsuki T., Abdollahipour R., Lewthwaite R., Wulf G., 2019 ** 1st experiment excluded (does not comply with PICO)	2		(48 h)	Putting accuracy error (the lower the better)	Randomly assigned	36	26.1	Yes	Novice	Lab	Cm	Golf putting	p = 0.100
		3			Throwing accuracy (the higher the better)		32	23.2					Throwing task	p = 0.005
9	Lin, C.H.J., Yang, H.C., Knowlton, B.J., Wu A.D., Iacoboni M., Ye, Y.L., Huang, S.L., Chiang, M.C., 2018	1	Blocked	(3 days)	Response time (RT) (the lower the better)	Within participants cross-over design	26	23.3 ± 1.3	Yes	Novice	Lab	ms	Serial reaction time task (SRT)	p = 0.3
			Random											
10	Chalavi, S., Pauwels, L., Heise, K.F., Zivariadab, H., Maes, C., Puts, N.A.J., Edden, R.A.E., Swinnen, S.P., 2018	1/young	Random	(6 days)	Performance accuracy (average track deviation ATrD) (The lower the better)	Randomly assigned	32	21.8 ± 1.8 (18–27)	Yes	Novice	Lab	Error scores	Visuomotor task	
			Blocked											
		2/older	Random				28	66.5 ± 4.1 (60–74)	No					
			Blocked											
11	Aiken C.A., Genter A.M., 2018	1	Blocked	Immediate	General accuracy through ring score	Randomly assigned	24	20.08 (18–22)	Yes	Novice	Applied	Scores	Golf chip shot	
			Random											
12	Jimenez-Diaz J., Morera-Castro M., Salazar W., 2018	1/overarm throw		(5 days)	Performance accuracy (the higher the better)	Randomly assigned	34	20.32 ± 2.07	Yes	Novice	Applied	Points—TFMSA test of fundamental motor skills for adults	Throwing task	
		1/distance jump											Jump	
13	Kim, T., Chen, J., Verwey, W.B., Wright, D.L., 2018	1		(48 h )	Total time (TT) (The lower the better)	N/A	24	> 18 (undergraduate students)	Yes	Novice	Lab	ms	Discrete sequence production task (typing)	
14	Parab, S., Bose, M., Ganesan, S., 2018	1/pattern A		Immediate	Time variable (the lower the better)	Randomly assigned	120 in total	6.5 ± 0.7 (6–8)	Pupil	Novice	Applied	s	Single leg hopping	
				(24 h)										
				(7 days)										
				Immediate				8.5 ± 0.7 (8–10)						
				(24 h)										
				(7 days)										
				Immediate				10.5 ± 0.07 (10–12)						
				(24 h)										
				(7 days)										
				Immediate	Error variable (the lower the better)		40	6.5 ± 0.7 (6–8)				Scores		
				(24 h)										
				(7 days)										
				immediate			40	8.5 ± 0.7 (8–10)						
				(24 h)										
				(7 days)										
				Immediate			40	10.5 ± 0.07 (10–12)						
				24 h										
				(7 days)										
		1/pattern B		Immediate	Time variable		40	6.5 ± 0.7 (6–8)				s		
				(24 h)										
				(7 days)										
				Immediate			40	8.5 ± 0.7 (8–10)						
				(24 h)										
				(7 days)										
				Immediate			40	10.5 ± 0.07 (10–12)						
				(24 h)										
				(7 days)										
				Immediate	Error variable		40	6.5 ± 0.7 (6–8)				Scores		
				(24 h)										
				(7 days)										
				Immediate			40	8.5 ± 0.7 (8–10)						
				(24 h)										
				(7 days)										
				Immediate			40	10.5 ± 0.07 (10–12)						
				(24 h)										
				(7 days)										
		1/pattern C		Immediate	Time variable		40	6.5 ± 0.7 (6–8)				s		
				(24 h)										
				(7 days)										
				Immediate			40	8.5 ± 0.7 (8–10)						
				(24 h)										
				(7 days)										
				Immediate			40	10.5 ± 0.07 (10–12)						
				(24 h)										
				(7 days)										
				Immediate	Error variable		40	6.5 ± 0.7 (6–8)				Scores		
				(24 h)										
				(7 days)										
				Immediate			40	8.5 ± 0.7 (8–10)						
				(24 h)										
				(7 days)										
				Immediate			40	10.5 ± 0.07 (10–12)						
				(24 h)										
				(7 days)										
15	Moretto, N.A., Marcori, A.J., Okazaki, V.H.A., 2018	1	Random	(7 days)	Shooting accuracy (the higher the better)	Subjects were divided into 2 groups	32	18–32	Yes	Novice	Applied	Point score	Sports riffle shooting	
			Blocked											
16	Broadbent, D.P., Causer, J., Williams, A.M., Ford, P.R., 2017 ***experiment 1st excluded, in 2nd experiment only blocked and random group results were included	2		Immediate	Decision time (ms) (DT) (The lower the better)	Randomly divided	28	20.8	Yes	Novice	Lab	ms	Primary anticipation task	
17	Fazeli, D., Taheri, H., Saberi, K. A.,2017	1	Random	(7 days)	Putting accuracy (the lower the better)	Randomly assigned	20	27.4 ± 4.6	Yes	Novice	Applied	cm	Golf putting	p < 0.05
18	Shewokis, P.A., Shariff, F.U., Liu, Y., Ayaz, H., Castellanos, A., Lind, D.S., 2017	1		(72 h)	Total time (s) (the lower the better)	Randomly assigned	10	> 18 y.o. (medical students)	Yes	Novice	Applied	s	Camera navigation	
													Lifting & grasping	
													Fine dissection	
19	Porter, J.M., Beckerman, T., 2016	1		(24 h)	Total time on target (the higher the better)	Randomly placed	52	20.7	Yes	Novice	Lab	s	Rotary pursuit tracking	
20	(A) Frömer, R., Stürmer, B., Sommer, W., 2016	1		(1 week)	Hit rates/accuracy scores (the higher the better)	Randomly assigned	120	25 ± 6	Most, but not all of the participants were students	Familiarized with throwing, but not experts	Lab	Points	Virtual dart throwing	
					Reaction time (RT) (the lower the better)							ms		
21	Cheong, J.P.G., Lay, B., Razman, R., 2016	1		(1 week)	Dribble speed (the lower the better)	N/A	44	21.56 ± 1.23 (19.17–26.67)	Yes	Novice	Applied	s	Field hockey skills	
				(3 weeks)										
				(1 week)	Push accuracy (the higher the better)							Points		
				(3 weeks)										
				(1 week)	Push speed (the higher the better)							km/h		
				(3 weeks)										
22	Broadbent, D.P., Causer, J., Ford P.R., Williams, A.M., 2015	1		(1 week)	Response accuracy	Randomly assigned	18	13.05 ± 1.6	Pupil	Intermediate (blocked: 5.3 ± 2.2 y., random: 5.9 ± 3.1)	Lab	Percentage	Tennis shots	
23	Rivard, J.D., Vergis, A.S., Unger, B.J., Gillman, L.M., Hardy, K.M., Park, J., 2015	1/peg transfer		(6 weeks)	Number of movements	Randomized	27	B = 28.5 ± 3.6, R = 28.5. ± 4.1	Yes	Novice	Applied		Laparoscopic skills	
					Median path-length travelled							m		
					Time to complete the task							s		
					FLS points							Points		
		1/pattern cutting			Number of movements									
					Median path-length travelled							m		
					Time to complete the task							s		
					FLS points							Points		
		1/ligating loop			Number of movements									
					Median path-length travelled							m		
					Time to complete the task							s		
					FLS points							Points		
		1/IC suturing			Number of movements									
					Median path-length travelled							m		
					Time to complete the task							s		
					FLS points							Points		
24	Naimo, M.A., Zourdos, M.C., Wilson, J.M., Kim, J.S., Ward, E.G., Eccles, D.W., Panton, L.B., 2013	1		(9 days)	Push accuracy (the higher the better)	Stratified based on gender etc	16	B = 20.55 ± 2.4, R = 20.55 ± 1.3	N/A	Novice	Applied		Bench pressing	
25	Saemi, E., Porter, J.M., Varzaneh, A.G., Zarghami, M., Shafinia, P., 2012	1		(24 h)	Push accuracy (the higher the better)	Randomly assigned	24	10.47 ± 0.77	Yes	Novice	Applied	Point scores	Overarm tennis ball throwing	
26	Lin, C.H.J., Chiang, M.C., Wu, A.D., Iacoboni, M., Udompholkul, P., Yazdanshenas, O., Knowlton, B.J., 2012	1/younger	Random testing	(5 days)	Push accuracy (the lower the better)	Within subject cross-over design	16	26.4 ± 3.1	Yes	Novice	Lab	ms	SRT finger tapping task	
			Blocked testing											
		1/older	Random testing		Serial reaction time (SRT) )the lower the better)		16	66.2 ± 4.7	No					
			Blocked testing											
27	Cheong, J.P.G., Lay, B., Grove, R.J., Medic, N., Razman, R., 2012	1/Indian dribble		(1 week)	Ball control	Randomly assigned	21	18 ± 0.3	Pre-university	Novice	Applied	Scores	Hockey skills	
					Movement form									
		1/push pass			Accuracy (the higher the better)									
					Speed							km/h		
					Movement form							Scores		
		3/hit			Accuracy (the higher the better)									
					Speed							km/h		
					Movement form							Scores		
28	(A) Porter, J.M., Magill,R.A., 2010****2nd experiment excluded due to missing data	1		(24 h )	Putting accuracy (mean error scores) (the lower the better)	Randomly assigned	40	> 18	Yes	Novice	Applied	Error scores	Golf putting	
29	Bertollo, M., Berchicci, M., Carraro, A., Comani, S., Robazza, C., 2010	1		(3 weeks)	Spatial accuracy (the higher the better)	Randomly assigned	40	15.8 ± 1.3	Pupils	Novice	Applied	Scores	Dance steps	
30	Simon, D.A., Lee, T.D., Cullen, J.D., 2008	1		(24 h )	% absolute constant error (the lower the better)	Randomly assigned	24	20.7 ± 2.4	Yes	Novice	Lab	%	Sequential key pressing	
31	Simon, D.A., 2007*****data of four participants removed from the study	1	Blocked	(24 h)	% absolute constant error (%CE) (The lower the better)	Randomly assigned	39—4 participants' data removed from the study	19.40	Yes	Novice	Lab	%	Key pressing sequences	
32	Zetou E., Michalopoulou M., Giazitzi K., Kioumourtzoglou E., 2007	1/set		(2 weeks)	AAPHER evaluation scores (the higher the better)	Randomly assigned	26	12.4 ± 1.2	Pupils	Novice	Applied	Points (AAPHER) out of 50 points	Volleyball skills	
		1/pass												
		1/service												
33	Russell, D.M., Newell, K.M., 2007	1/1. block the first block (out of 3 testing blocks) has been chosen for retention to eliminate further learning benefits in 2nd and 3rd retention blocks	Blocked	(24 h )	Total time (the lower the better)	Randomly assigned	48	21.9 ± 3.5	Yes	Novice	Lab	ms	Rapid sequential aiming task	
			Random											
			Blocked		Reaction time									
			Random											
			Blocked		Movement time									
			Random											
			Blocked		Error									
			Random											
34	Ste-Marie, D.M., Clark, S.E., Findlay, L.C., Latimer, A.E., 2004 ***** Retention data not possible to obtain from the Ist and the 2nd experiments	3		Immediate	Scoring system (the higher the better)	Randomly assigned	68	6.42 ± 0.40 (5,5–7)	Pupils	Novice	Applied		Handwriting	
35	Moreno, F.J., Ávila, F., Damas, J., García, J.A., Luis, V., Reina, R., Ruíz, A., 2003	1/darts	Blocked	(48 h)	Accuracy of the impact on the target (the higher the better)	Randomly assigned	35	19.9 ± 0.8	Yes	Novice	Lab		Darts throwing	
			Random											
		1/side ball	Blocked											
			Random											
		1/low ball	Blocked											
			Random											
		1/darts	Blocked	(4 weeks)										
			Random											
		1/side ball	Blocked											
			Random											
		1/low ball	Blocked											
			Random											
		1/darts	Blocked	(8 weeks)										
			Random											
		1/side ball	Blocked											
			Random											
		1/low ball	Blocked											
			Random											
36	Vera, J.G., Montilla, M.M., 2003	1		(2 weeks)	Throwing accuracy (The higher the better)	Assigned based on pretest results	71	M = 6	Pupils	Novice	Applied	Scores	Throwing task	
37	Smith, P.J.K., 2002	1/5 s task duration		(24 h)	Total time on target (the higher the better)	Randomly assigned	48	M = 20.4; (18–29)	Yes	Novice	Lab	s (time on a target)	Pursuit rotor	
		1/ 20 s task duration												
38	Li, Y., Lima, R.P., 2002	11 yd	Blocked	(24 h)		Randomly assigned	38	22.4 ± 2.9 (18–29)	N/A	Novice	Applied	Scores	Passing a soccer ball to the target	
			Random											
		17 yd	Blocked											
			Random											
		22 yd	Blocked											
			Random											
		11.yd	Blocked	Immediate										
			Random											
		17 yd	Blocked											
			Random											
		22 yd	Blocked											
			Random											
39	Shea, C.H., Lai, Q., Wright, D.L., Immink, M., Black, C., 2001******* The 2nd experiment results were excluded due to groups characteristics	1		(24 h)	Relative-timing error (the lower the better)	Randomly assigned	20	Students > 18	Yes	Novice	Lab		Sequential key pressing task	
40	Li, Y., Wright, D.L., 2000	1		(24 h)	Mean absolute constant error (ACE) (the lower the better)	Randomly assigned	28	19–27	Yes	Novice	Lab	ms	Key-pressing sequences	
					Variable error (VE)									
41	Brady, F., 1997	1		(1 week)	Total number of shots (the lower, the better)	(Based on classes)	36	20.4 ± 3.1 (19–27)	Yes	Novice	Applied	Number of shots	Golf skills	
42	Pollatou, E., Kioumourtzoglou, E., Agelousis, N., Mavromatis, G.,1997	1/throwing		(1 week)	Error scores (The lower, the better)	Randomly assigned	42	> 18	Yes	Novice	Lab	Accuracy scores	Throwing/kicking	
		1/kicking												
		1/throwing		(24 h)										
		1/kicking												
43	Goodwin, J.E., Meeuwsen, H.J., 1996******** 2 groups were chosen out of 3 groups	1/distance 2.43 m		(24 h)	Absolute error	Randomly assigned	20	26.2 ± 8.0	Yes	Novice	Applied	m	Golf putting	
		1/distance 3.95 m												
		1/distance 5.47 m												
44	Smith, P.J., Davies, M., 1995	1/full Pawlata roll	Preferred side	(1 week)		Randomly assigned	16	22.06 (18–30)	Yes	Novice	Applied	Scores (1–3)	Pawlata roll	
			Non-preferred side											
45	Del Rey, P., Liu, X., Simpson, K.J., 1994*********	1/1. trial the first trial (out of 3 testing trials) has been chosen for retention to eliminate further learning benefits in 2nd and 3rd retention trials)		Immediate	Reaction time (RT) (the lower the better)	Randomly assigned	30	22 ± 3.18	Yes	Novice	Lab	ms	Key pressing task	
46	Wright, D.L., Li, Y., Whitacre, C.,1992	1		(3 weeks)	Movement time	N/A	20	> 18 (undergraduate students)	Yes	Novice	Lab	s	Sequential key presses	
47	Bortoli, L., Robazza, C., Durigon, V., Carra, C.,1992	1/bump		(1 week)	Scores (the higher the better)	Randomly assigned	26	14.6 ± 0.7	Pupils	Novice	Applied	Scores	Volleyball skills (bump, volley, serve)	
		1/volley												
		1/serve												
48	French, K.E., Rink, J.E., Werner, P.H., 1990********** out of 3 groups 2 groups were chosen	1/pass		(48 h)	Success rates (the higher the better)	Randomly assigned	93	18 < highschool students	Pupils	Novice	Applied	Success rates (%)	Volleyball pass	p > 0.05
		1/set											Volleyball set	
		1/serve											Volleyball serve	
49	Porter, J. M., Landin, D., Hebert, E.P., Baum, B., 2007 ***********out of 3 groups, 2 groups were chosen	1	Blocked	(3 days)	Scores (the higher the better)	Randomly assigned	14	19.5 ± 1.25	Yes	Novice	Lab	Score (points)	Two golf skills—putt and pitch	
					Trial scores									
					Movement pattern (form) scores									
			Random		Trial scores									
					Movement pattern (form) scores									
50	Pasand, F., Fooladiyanzadeh, H, Nazemzadegan, G., 2016	1		(48 h)	Accuracy score—volleyball skills (the higher the better)	Randomly assigned	30	22.5 ± 1.7	Yes	Novice	Applied	Score	Volleyball skills	
51	Waqqash, E., Low, J., 2015	1		Immediate	Squash shot accuracy	Randomly assigned	8	> 18 (university students)	Yes	Novice	Applied	Score (points)	Squash shot (backhand/forehand)	
52	Tsutsui, S.,Satoh M., Yamamoto, K. 2013	1	Random	(24 h)	Successful trials	Randomly assigned	20	15–22	NA	Experienced	Applied	Number of successful trials	Baseball skills	
										Novice				
53	Wong, A.W.K., Whitehill, T.L.; Ma, E.P.M.; Masters, R., 2013************ out of 4 groups, 2 groups were chosen	1	Blocked	Immediate	Mean duration difference (target 2500 ms vs 3500 ms)	Randomly assigned	20	20.8 ± 1.6	Yes	Novice	Lab	RMSE (ms)	Novel speech task secondary key pressing task (transfer)	
				(48 h)										
54	(B) Porter, J.M., Saemi, E. 2010*************out of 3 groups 2 groups were chosen	1	Blocked	Immediate	Absolute error (the lower the better)	Randomly assigned	30	> 18 (undergraduate students)	Yes	Moderately skilled (participants had passed a basketball activity course they were considered moderately skilled at passing a basketball, which involved skills they were taught in their respective basketball course. None of the participants played college or professional basketball; however some participants acknowledged that they did play basketball recreationally from time to time	Applied	Absolute error score	Three basketball passes	
				(48 h)										
55	Beik, M.; Taheri, H.; Saberi K.A., Ghoshuni M., 2020 **************out of 6 groups 4 groups were chosen	1	Blocked/similar	(24 h)	Total error (the lower the better)	Randomly assigned	40	67.68 ± 3.95	No	Novice	Lab	ms	Sequential motor task	
			Dissimilar											
56	Green, S. & Sherwood, D.E. 2000	1		Immediate	Overall error (temporal performance) (the lower the better)	Randomly assigned	32	18–23	Yes	Novice	Lab	Error score	Rapid reversal movement	
				(24 h)										
57	Jeon, M.J.; Jeon, H.S.; Yi, C.H.; Kwon, O.Y.; You, S.H.; Park, J.H., 2020	1	Functional reach test (FRT)	(3 days)	Distance (the higher the better)	Randomly assigned	41	65–82 (B = 71.3 ± 4.2), (R = 72.8 ± 5,2)	No	Novice	Lab	cm	FRT	
			Timed up and go test (TUG)		Completion time (the lower the better)							s	TUG	
			Performance oriented mobility assessment (POMA)		Performance score (the higher the better)							Score	POMA	
			Wii-soccer heading		Score								Wii-soccer heading	
			Wii-ski slalom										Wii-ski slalom	
			Wii-snowboard slalom										Wii-snowboard slalom	
			Functional reach test (FRT)	(7 days)	Distance (the higher the better)							cm	FRT	
			Timed up to go test (TUG)		Completion time (the lower the better)							s	TUG	
			Performance oriented mobility assessment (POMA)		Performance score (the higher the better)							Score	POMA	
			Wii-soccer heading		Score								Wii-soccer heading	
			Wii-ski slalom										Wii-ski slalom	
			Wii-snowboard slalom										Wii-snowboard slalom	
58	Sherwood D.E., 1996 *************** The 2nd experiment results were excluded as they were considered as transfer results	1		Immediate	Mean constant error in time to reversal (the lower the better)	Quasi-randomly assigned	24	21.2 ± 2.3	Yes	Novice	Lab	ms	Rapid lever reversal movement	p > 0.05
				(24 h)										p > 0.05
				Immediate	Mean spatial absolute constant error (the lower the better)							Degrees (spacial)		
				(24 h)										
59	Kaipa, R.; Kaipa, R.M., 2018	1		Immediate	Absolute error: EMG-amplitude of lip muscle contraction	Randomly assigned	15	21.93 ± 1.75 (2–27)	Yes	Novice	Lab	MVC, absolute error score	Oral motor tracking task	
				(4 days)			15	21.27 ± 1.33 (19–24)						

*Schorn, J.M., Knowlton, B.J., 2021, 2nd Experiment—Experiment II focused on transfer and not obtained information from authors regarding number of participants in particular groups (BB, BI, II, IB).

**Chua, L.K., Dimapilis, M.K., Iwatsuki, T., Abdollahipour, R., Lewthwaite, R., Wulf G., 2019, Experiment 1—excluded: constant or variable practice group instead of random/blocked groups. Variable group participants threw from all three distances (4, 5, and 6 m) during practice. The order of distances was pre-determined and quasi-random, with the constraint that each distance occurred 20 times. Constant group participants were divided into three subgroups, and each subgroup threw from one of the distances (4 m, 5 m, or 6 m) for a total of 60 practice trials.

***Broadbent, D.P., Causer, J., Williams, M.A., Ford, P.R., 2017, The first experiment was not considered for meta-analysis as it did not comply with PICO (dual task procedure was applied), in the 2nd task the blocked and random groups (without the Stroop effect) were chosen. (*Novice participants were divided into blocked, random, blocked-Stroop(BStroop), and random-Stroop (RStroop) groups*. Additionally—however tasks were purely cognitive, they formed (were part of) motor tasks. Anticipation is an important part of each motor task (e.g. where will be opponent in a second and where should a person pass the ball).

****(A) Porter, J.M., Magill, R.A., 2010, 2nd Experiment—Not obtained information from authors regarding results.

*****Simon, D.A., 2007, Data of four participants removed from the study.

******Ste-Marie, D.M., Clark, S.E., Findlay L.C., Latimer, A.E., 2004*, There was no possibility to obtain retention data from experiments 1st and the 2nd.

*******Shea, C.H., Lai, Q., Wright, D.L., Immink, M., Black, C., 2001, Not obtained nonsignificant results from authors regarding absolute timing outcome measure. The 2nd experiment results were excluded due to groups characteristics (group of ratio-feedback /blocked and random and group of segment-feedback/blocked and random).

********Goodwin, J.E., Meeuwsen, H.J., 1996, Out of 3 groups (Random, Blocked-Random, or Blocked practice condition) 2 groups were chosen (Random and Blocked practice condition).

*********Del Rey, P., Liu, X., Simpson, K.J.,1994, Out of 5 groups (Control, BL-without, BL-18, BL-36, Random) 2 groups (Random, BL-without) were chosen as they meet the PICO criteria.

**********French, K.E., Rink, J.E., Werner, P.H.,1990, Out of 3 groups (Random, Random-Blocked, or Blocked practice), 2 groups were chosen (random and blocked).

***********Porter, J. M., Landin, D., Hebert, E.P., Baum, B., 2007, Out of 3 groups (High contextual Interference, Moderate Contextual Interference, Low Contextual Interference), 2 groups were chosen (High CI and Low CI).

************Wong, AWK; Whitehill, TL; Ma, EPM; Masters, R, 2013, Out of 4 groups (Blocked only, Random only, Blocked-then-Random, and Random-then-Blocked) 2 groups were chosen: Blocked and Random.

*************(B) Porter JM , E Saemi, 2010Out of 3 groups (Blocked, Random, or Increasing-CI practice schedule) 2 groups were chosen (Blocked and Random).

*************Beik, M.; Taheri, H.; Saberi Kakhki, A.; Ghoshuni, M., 2020, Out of 6 groups ( blocked-similar (BS), algorithm-similar (AS), random-similar (RS), blocked-dissimilar (BD), algorithm dissimilar (AD), or random-dissimilar (RD) 4 groups were chosen (blocked-similar, random-similar, blocked-dissimilar, random-dissimilar).

**************Sherwood, D.E., 1996, The 2nd experiment results were excluded as they were considered as transfer results *In addition, a 30° amplitude was added during a retention test as a novel transfer amplitude.*

### Reasons for exclusion of individual experiments or particular groups of participants

From some studies not all experiments were included, only those that complied with the inclusion criteria. The reasons for exclusion are provided below.

In the study by Schorn and Knowlton^4^, two experiments were performed. Only the first one was included in the present review. The second experiment examined the transfer. Furthermore, the authors did not provide details regarding a number of participants in each of the four groups. Neither were these details provided in the paper nor by the authors in e-correspondence. Another study consisting of more than one experiment was an article by Ste-Marie and colleagues^46^, where CI effect on learning handwriting skills in young participants was examined. Only results of (immediate) retention testing were available from the third experiment, as the results available in the text regarding the second experiment mainly focused on transfer. It was not possible to obtain the results from the text in the first experiment.

Of the two experiments conducted in the study by Porter and Magill^47^, results from the first experiment were possible to obtain. In case of the study by Shea and colleagues^48^ we were not able to obtain the absolute timing error results (reported as non-significant) from the authors. The second experiment’ results of the mentioned study were not included due to group characteristics (group of ratio-feedback/blocked and random and group of segment-feedback/blocked and random) not compliant with PICO.

Chua and colleagues conducted three experiments, of which two (the second and the third one) were included in the present study. The first experiment was not included as it described constant practice group instead of blocked practice: Constant group participants were divided into three subgroups, and each subgroup threw from one of the distances (4 m, 5 m, or 6 m) for a total of 60 practice trials^49^. The study of Broadbent and colleagues^50^ consisted of two experiments. In the first experiment, the authors applied a dual-task procedure. The second one, beside CI effect, involved Stroop procedures which resulted in four groups (blocked-Stroop, blocked, random-Stroop, random). Hence, the results of two groups (random and blocked) were chosen for analysis. Sherwood^51^ conducted two experiments utilizing a rapid aiming task. However, the results of the second experiment were excluded from the present review, as these were considered to be transfer results.

In an experiment by Beik and colleagues^52^, participants were randomly assigned to six groups: blocked-similar, algorithm-similar, random-similar, blocked-dissimilar, algorithm dissimilar, or random-dissimilar. According to PICO, retention results of four groups (blocked-similar, random-similar, blocked-dissimilar, random-dissimilar) were considered applicable for the present review. Consistently, out of the four groups (blocked only, random only, blocked-then-random, and random-then-blocked) involved in the study by Wong and colleagues^53^, two groups were chosen for the purpose of the current review: blocked schedule group and random schedule group.

A study by Porter and colleagues^54^ described acquisition and retention of participants randomly assigned to three practice groups: high, moderate, and low CI group. For the present review, the retention results of two groups (high CI and low CI) were extracted. The following study of Porter^55^ consisted of three groups: blocked, random, and increasing-CI practice schedules. Only blocked and random schedule groups were included.

In the study by Del Rey and colleagues^56^, CI effect was examined in key-pressing task performed by five practice groups. The groups were different in terms of the administered amount of CI and the presence (or absence) of retroactive inhibition. Retention results of two groups (random, blocked-without retroactive inhibition) were chosen for the purpose of the present study.

French and colleagues^57^ in a study on CI in learning volleyball skills, randomly assigned participants to three acquisition groups: random, random-blocked, or blocked practice. The random and the blocked practice groups were included in the present review. Similarly, in an article by Goodwin and Meeuwsen on CI effect in learning golf skills^58^, three groups of participants were tested: learning in random, blocked-random, or blocked practice condition. Consistently, only blocked and random practice schedule groups were included in the current review.

### Results of quality assessment of included studies

The results of the methodological assessment of the studies included in our systematic review are highlighted in Table 2. Only three articles presented moderate^4,46^ or high^59^ methodological quality according to the Quality Assessment Tool for Quantitative Studies^44^. The primary studies failed mainly on the following criteria: 44 articles scored weak rating in the *Selection Bias* section, in *Withdrawals And Drop-outs* section 50 studies scored weak rating. Such relatively strict evaluation records could be explained by the fact that two weak ratings were enough to automatically determine a weak classification of an article in its global rating for all six components of the checklist.

**Table 2:** Quality assessment of included studies. Q1, Q2, ... - question number accroding to the Quality Assessment Tool for Quantitative Studies

	Authors	Title	Year	Journal title	# Experiment	Quality assessment																	
						Selection bias			Study design		Confounders			Blinding			Data collection methods			Withdrawals and drop-outs			Global rating
						Q1	Q2	Rate	Q1	Rate	Q1	Q2	Rate	Q1	Q2	Rate	Q1	Q2	Rate	Q1	Q2	Rate	
1	Thomas, J.L., Fawver, B., Taylor, S., Miller M.W., Williams, A.M., Lohse, K.R	Using error-estimation to probe the psychological processes underlying contextual interference effects	2021	*Human Movement Science*		2	1	2	2	1	2	N/A	1	3	3	3	1	1	1	2	4	3	3
2	Beik, M., Fazeli, D	The effect of learner-adapted practice schedule and task similarity on motivation and motor learning in older adults	2021	*Psychology of Sport and Exercise*		4	1	3	2	1	2	N/A	1	3	2	2	1	1	1	2	4	3	3
3	Immink, M.A., Pointon, M., Wright, D.L., Marino, F.E	Prefrontal Cortex Activation During Motor Sequence Learning Under Interleaved and Repetitive Practice: A Two-Channel Near-Infrared Spectroscopy Study	2021	*Frontiers in Human Neuroscience*		4	1	3	2	1	3	N/A	3	3	2	2	1	1	1	2	4	3	3
4	Kim, T., Kim, H., Wright, D.L	Improving consolidation by applying anodal transcranial direct current stimulation at primary motor cortex during repetitive practice	2021	*Neurobiology of Learning and Memory*		3	1	3	2	1	2	N/A	1	3	2	2	1	1	1	2	4	3	3
5	Schorn, J.M., Knowlton, B.J	Interleaved practice benefits implicit sequence learning and transfer	2021	*Memory and Cognition*	1. Experiment	3	1	3	2	1	2	N/A	1	3	2	2	1	1	1	1	1	1	2
6	Beik M., Taheri H., Ghoshuni M	Algorithm-Based Practice Schedule and Task Similarity Enhance Motor Learning in Older Adults	2021	*Journal of Motor Behavior*		3	1	3	2	1	2	N/A	1	3	2	2	1	1	1	2	4	3	3
7	Porter C., Greenwood D., Panchuk D., Pepping G.-J.,	Learner-adapted practice promotes skill transfer in unskilled adults learning the basketball set shot	2020	*European Journal of Sport Science*		4	1	3	2	1	3	N/A	3	3	2	2	3	3	3	2	4	3	3
8	Chua L.-K., Dimapilis M.K., Iwatsuki T., Abdollahipour R., Lewthwaite R., Wulf G.,	Practice variability promotes an external focus of attention and enhances motor skill learning	2019	*Human Movement Science*	2. Experiment	3	1	3	2	1	3	N/A	3	3	2	2	1	1	1	3	4	3	3
					3. Experiment	3	1	3	2	1	3	N/A	3	1	2	2	1	1	1	2	4	3	
9	Lin C.-H.J., Yang H.-C., Knowlton B.J., Wu A.D., Iacoboni M., Ye Y.-L., Huang S.-L., Chiang M.-C.,	Contextual interference enhances motor learning through increased resting brain connectivity during memory consolidation	2018	*NeuroImage*		4	1	3	2	1	2	N/A	1	3	3	3	1	1	1	2	4	3	3
10	Chalavi S., Pauwels L., Heise K.-F., Zivariadab H., Maes C., Puts N.A.J., Edden R.A.E., Swinnen S.P.,	The neurochemical basis of the contextual interference	2018	*Neurobiology of Aging*		4	1	3	2	1	2	N/A	1	3	2	2	1	1	1	2	4	3	3
11	Aiken C.A., Genter A.M.,	The effects of blocked and random practice on the learning of three variations of the golf chip shot	2018	*International Journal of Performance Analysis in Sport*		3	1	3	2	1	3	N/A	3	3	2	2	1	1	1	2	4	3	3
12	Jimenez-Diaz J., Morera-Castro M., Salazar W.,	The contextual interference effect on the performance of fundamental motor skills in adults	2018	*Human Movement*		4	1	3	2	1	2	N/A	1	3	3	3	1	1	1	1	1	1	3
13	Kim T., Chen J., Verwey W.B., Wright D.L.,	Improving novel motor learning through prior high contextual interference training	2018	*Acta Psychologica*		3	1	3	N/A	N/A	3	N/A	3	3	2	2	1	1	1	2	4	3	3
14	Parab S., Bose M., Ganesan S.,	Influence of random and blocked practice schedules on motor learning in children aged 6–12 years	2018	*Critical Reviews in Physical and Rehabilitation Medicine*		2	1	1	2	1	2	N/A	1	3	3	3	1	1	1	2	4	3	3
15	Moretto N.A., Marcori A.J., Okazaki V.H.A.,	Contextual interference effects on motor skill acquisition, retention and transfer in sport rifle shooting	2018	*Human Movement*		4	1	3	N/A	N/A	3	N/A	3	3	3	3	1	2	2	2	4	3	3
16	Broadbent D.P., Causer J., Mark Williams A., Ford P.R.,	The role of error processing in the contextual interference effect during the training of perceptual-cognitive skills	2017	*Journal of Experimental Psychology: Human Perception and Performance*	2. Experiment	4	1	3	2	1	2	N/A	1	3	3	3	1	1	1	2	4	3	3
17	Fazeli D., Taheri H., Saberi Kakhki A.,	Random Versus Blocked Practice to Enhance Mental Representation in Golf Putting	2017	*Perceptual and Motor Skills*		4	1	3	2	1	3	N/A	3	3	3	3	1	1	1	2	4	3	3
18	Shewokis P.A., Shariff F.U., Liu Y., Ayaz H., Castellanos A., Lind D.S.,	Acquisition, retention and transfer of simulated laparoscopic tasks using fNIR and a contextual interference paradigm	2017	*American Journal of Surgery*		3	1	3	2	1	2	N/A	1	3	3	3	1	1	1	1	1	1	3
19	Porter J.M., Beckerman T.,	Practicing with gradual increases in contextual interference enhances visuomotor learning	2016	*Kinesiology*		3	1	3	2	1	3	N/A	3	3	2	2	3	3	3	2	4	3	3
20	Frömer R., Stürmer B., Sommer W.,	(Don't) Mind the effort: Effects of contextual interference on ERP indicators of motor preparation	2016	*Psychophysiology*		3	1	3	2	1	3	N/A	3	3	3	3	1	1	1	2	4	3	3
21	Cheong J.P.G., Lay B., Razman R.,	Benefit of interleaved practice of motor skills is associated with changes in functional brain network topology that differ between younger and older adults	2016	*Neurobiology of Aging*		3	1	3	N/A	N/A	3	N/A	3	3	3	3	1	1	1	2	4	3	3
22	Broadbent D.P., Causer J., Ford P.R., Williams A.M.,	Contextual interference effect on perceptual-cognitive skills training	2015	*Medicine and Science in Sports and Exercise*		3	1	3	2	1	2	N/A	1	3	3	3	1	1	1	3	4	3	3
23	Rivard J.D., Vergis A.S., Unger B.J., Gillman L.M., Hardy K.M., Park J.,	The effect of blocked versus random task practice schedules on the acquisition and retention of surgical skills	2015	*American Journal of Surgery*		2	1	2	1	1	2	N/A	1	3	3	3	1	1	1	2	4	3	3
24	Naimo M.A., Zourdos M.C., Wilson J.M., Kim J.-S., Ward E.G., Eccles D.W., Panton L.B.,	Contextual interference effects on the acquisition of skill and strength of the bench press	2013	*Human Movement Science*		2	1	2	2	1	2	N/A	1	2	3	2	1	1	1	1	1	1	1
25	Saemi E., Porter J.M., Varzaneh A.G., Zarghami M., Shafinia P	Practicing along the contextual interference continuum: A comparison of three practice schedules in an elementary physical education setting	2012	*Kinesiology*		2	1	2	2	1	3	N/A	3	3	3	3	1	3	2	2	4	3	3
26	Lin C.-H.J., Chiang M.-C., Wu A.D., Iacoboni M., Udompholkul P., Yazdanshenas O., Knowlton B.J.,	Age related differences in the neural substrates of motor sequence learning after interleaved and repetitive practice	2012	*NeuroImage*		3	1	3	2	1	2	N/A	1	3	3	3	1	3	2	2	4	3	3
27	Cheong J.P.G., Lay B., Robert Grove J., Medic N., Razman R.,	Practicing field hockey skills along the contextual interference continuum: A comparison of five practice schedules	2012	*Journal of Sports Science and Medicine*		3	1	3	2	1	3	N/A	3	3	3	3	1	1	1	2	4	3	3
28	Porter J.M., Magill R.A.,	Systematically increasing contextual interference is beneficial for learning sport skills	2010	*Journal of Sports Sciences*	1. Experiment	2	1	2	2	1	3	N/A	3	3	3	3	3	3	3	2	4	3	3
29	Bertollo M., Berchicci M., Carraro A., Comani S., Robazza C.,	Blocked and random practice organization in the learning of rhythmic dance step sequences	2010	*Perceptual and Motor Skills*		3	1	3	2	1	3	N/A	3	3	3	3	1	3	2	2	4	3	3
30	Simon D.A., Lee T.D., Cullen J.D.,	Win-shift, lose-stay: Contingent switching and contextual interference in motor learning	2008	*Perceptual and Motor Skills*		3	1	3	2	1	3	N/A	3	3	2	2	1	3	2	2	4	3	3
31	Simon D.A.,	Contextual interference effects with two tasks	2007	*Perceptual and Motor Skills*		3	3	3	2	1	3	N/A	3	3	3	3	1	3	2	2	4	3	3
32	Zetou E., Michalopoulou M., Giazitzi K., Kioumourtzoglou E.,	Contextual interference effects in learning volleyball skills	2007	*Perceptual and Motor Skills*		2	1	2	2	1	3	N/A	3	3	2	2	1	1	1	2	4	3	3
33	Russell D.M., Newell K.M.,	How persistent and general is the contextual interference effect?	2007	*Research Quarterly for Exercise and Sport*		3	1	3	2	1	3	N/A	3	3	2	2	1	1	1	2	4	3	3
34	Ste-Marie D.M., Clark S.E., Findlay L.C., Latimer A.E.,	High Levels of Contextual Interference Enhance Handwriting Skill Acquisition	2004	*Journal of Motor Behavior*	3. Experiment	2	1	2	2	1	3	N/A	3	2	3	2	3	3	2	1	1	1	2
35	Moreno F.J., Ávila F., Damas J., García J.A., Luis V., Reina R., Ruíz A.,	Contextual interference in learning precision skills	2003	*Perceptual and Motor Skills*		3	1	3	2	1	3	N/A	3	3	3	3	3	3	2	2	4	3	3
36	Vera J.G., Montilla M.M.,	Practice schedule and acquisition, retention, and transfer of a throwing task in 6-yr.-old children	2003	*Perceptual and Motor Skills*		2	1	2	2	1	2	N/A	1	3	3	3	3	3	3	2	4	3	3
37	Smith P.J.K.,	Task duration in contextual interference	2002	*Perceptual and Motor Skills*		3	1	3	2	1	3	N/A	3	3	2	2	1	3	2	2	4	3	3
38	Li Y., Lima R.P.,	Rehearsal of task variations and contextual interference effect in a field setting	2002	*Perceptual and Motor Skills*		3	4	3	2	1	3	N/A	3	3	3	3	3	3	3	2	4	3	3
39	Shea C.H., Lai Q., Wright D.L., Immink M., Black C.,	Consistent and variable practice conditions: Effects on relative and absolute timing	2001	*Journal of Motor Behavior*	1. Experiment	3	1	3	2	1	3	N/A	3	3	2	2	1	3	2	2	4	3	3
40	Li Y., Wright D.L.,	An assessment of the attention demands during random- and blocked-practice schedules	2000	*Quarterly Journal of Experimental Psychology Section A: Human Experimental Psychology*		3	1	3	2	1	3	N/A	3	3	3	3	1	3	2	2	4	3	3
41	Brady F.,	Contextual interference and teaching golf skills	1997	*Perceptual and Motor Skills*		2	1	2	2	1	3	N/A	3	1	2	2	1	3	2	2	4	3	3
42	Pollatou E., Kioumourtzoglou E., Agelousis N., Mavromatis G.,	Contextual interference effects in learning novel motor skills	1997	*Perceptual and Motor Skills*		3	1	3	2	1	3	N/A	3	3	3	3	1	3	2	2	4	3	3
43	Goodwin J.E., Meeuwsen H.J.,	Investigation of the contextual interference effect in the manipulation of the motor parameter of over-all force	1996	*Perceptual and Motor Skills*		3	1	3	2	1	3	N/A	3	3	3	3	3	3	3	2	4	3	3
44	Smith P.J., Davies M.,	Applying contextual interference to the pawlata roll	1995	*Journal of Sports Sciences*		3	1	3	2	1	3	N/A	3	3	2	2	1	3	2	2	4	3	3
45	Del Rey P., Liu X., Simpson K.J.,	Does retroactive inhibition influence contextual interference effects?	1994	*Research Quarterly for Exercise and Sport*		3	1	3	2	1	2	N/A	1	3	3	3	1	1	1	2	4	3	3
46	Wright D.L., Li Y., Whitacre C.,	The contribution of elaborative processing to the contextual interference effect	1992	*Research Quarterly for Exercise and Sport*		3	1	3	2	1	3	N/A	3	3	2	2	1	3	2	2	4	3	3
47	Bortoli L., Robazza C., Durigon V., Carra C.,	Effects of contextual interference on learning technical sports skills	1992	*Perceptual and motor skills*		4	5	3	2	1	2	N/A	1	3	3	3	3	3	3	2	4	3	3
48	French K.E., Rink J.E., Werner P.H.,	Effects of contextual interference on retention of three volleyball skills	1990	*Perceptual and Motor Skills*		3	1	3	2	1	3	N/A	3	3	3	3	1	1	1	2	4	3	3
49	Porter J. M., Landin D., Herbert E.P., Baum B	The effects of three levels of contextual interference on performance outcomes and movement patterns in golf skills	2007	*International Journal of Sports Science & Coaching*		3	1	3	2	1	2	N/A	3	3	3	3	1	1	1	2	4	3	3
50	F Pasand, H Fooladiyanzadeh, Gholamhossien Nazemzadegan,	The Effect of Gradual Increase in Contextual Interference on Acquisition, Retention and Transfer of Volleyball Skills	2016	*International Journal of Kinesiology and Sport Science*		3	5	3	2	1	3	N/A	3	3	3	3	3	3	3	2	4	3	3
51	E Waqqash, J Low	Effects of contextual interference (CI) in basic squash shots practice	2015	*Malaysian Journal of Sport Science and Recreation*		3	1	3	2	1	3	N/A	3	3	3	3	3	3	3	2	4	3	3
52	Tsutsui S, M Satoh, K Yamamoto	Contextual Interference Modulated by Pitcher Skill Level	2013	*International Journal of Sport and Health Science*		3	1	3	2	1	3	N/A	3	3	3	3	1	3	2	2	4	3	3
53	Wong, AWK; Whitehill, TL; Ma, EPM; Masters, R	Effects of practice schedules on speech motor learning	2013	*International Journal of Speech-Language Pathology*		3	1	3	2	1	3	N/A	3	3	3	3	1	1	1	2	4	3	3
54	Porter JM , E Saemi	Moderately Skilled Learners Benefit by Practicing with Systematic Increases in Contextual Interference	2010	*International journal of coaching science*		3	1	3	2	1	3	N/A	3	3	3	3	3	1	2	2	4	3	3
55	Beik M; Taheri H; Saberi Kakhki A; Ghoshuni M	Neural Mechanisms of the Contextual Interference Effect and Parameter Similarity on Motor Learning in Older Adults: An EEG Study	2020	*Frontiers in Aging Neuroscience*		4	1	3	2	1	2	N/A	1	3	2	2	1	1	1	2	4	3	3
56	S Green, D.E. Sherwood	The benefits of random variable practice for accuracy and temporal error detection in a rapid aiming task	2000	*Research Quaterly for exercise and sport*		3	1	3	2	1	3	N/A	3	3	3	3	1	1	1	2	4	3	3
57	Jeon, MJ; Jeon, HS; Yi, CH; Kwon, OY; You, SH; Park, JH	Block and Random Practice: A Wii Fit Dynamic Balance Training in Older Adults	2020	*Research Quarterly for Exercise and Sport*		2	1	2	2	1	2	N/A	1	3	3	3	1	1	1	2	4	3	3
58	Sherwood D.E.,	The benefits of random variable practice for spatial accuracy and error detection in a rapid aiming task	1996	*Research Quarterly for Exercise and Sport*	1. Experiment	3	1	3	2	1	3	N/A	3	3	3	3	1	1	1	3	4	3	3
59	Kaipa, R; Kaipa, RM	Role of Constant, Random and Blocked Practice in an Electromyography-Based Oral Motor Learning Task	2018	*Journal of Motor Behavior*		3	1	3	2	1	3	N/A	3	3	3	3	1	1	1	2	4	3	3

Only studies rated strong and moderate should be included in the meta-analysis^26^. However, excluding studies rated as weak would make our analysis rather dubious (with only two studies included). Therefore, we have included fifty-four articles in the meta-analysis. Consistently, the impact of this decision on heterogeneity was considered.

### Findings

Only delayed retention testing results were included in the present meta-analysis, yielding 194 effect sizes. Outcomes from 54 studies were included in the meta-analysis, resulting in testing of 2068 participants, yielding 6183 measurements in total. A broad range of variables was involved: time (decision time, absolute error time, variable time, reaction time, response time, completion time), distance (accuracy error distance, absolute error distance, median pathway traveled), a number of performed movements, accuracy (accuracy scores, proficiency percentage). Outcome measures evaluating retention of the learned motor skills were presented in various units: meters, seconds, percentages, or scores.

In ten studies, the results of both testing procedures (immediate and delayed) were presented. In a study by Beik^60^, in addition to immediate testing, participants took part in delayed testing 24 h after their last acquisition session. In Kim's^61^ article, immediate retention testing was performed 6 h after acquisition, whereas the time interval between acquisition and delayed retention was 24 h. In a study by Porter^62^, immediate testing results and 7-days delayed testing results were presented. In an experiment by Kaipa et colleagues^63^, delayed retention testing was performed six days after acquisition. In an article by Parab and colleagues^64^, young participants took part in the following testing strategy: immediate testing, delayed testing trial (24 h after last acquisition), and finally—testing seven days after the last acquisition session. Wong and colleagues^53^ applied both testing procedures: immediate and delayed (48 h after the last acquisition session). Porter and Saemi^55^ described immediate and delayed (48 h) testing results. In an experiment by Li and Lima^65^, participants were involved in two testing procedures: immediate and delayed—24 h after the last acquisition session. A study by Sherwood^51^ described two testing strategies: immediate and 24 h delayed; similar procedures could be found in research by Green and Sherwood^66^.

Motor skills described in primary studies varied in many ways. They were presented in many configurations – gross motor skills or fine motor skills, continuous motor skills and discrete motor skills, open motor skills, and closed motor skills. Motor skills were associated with golf^47,49,54,58,67,68^, volleyball^57,69–71^, hockey^72,73^, soccer^65,74^, tennis^75^, darts throwing^76,77^, basketball^55,62^, dancing^78^, kicking^79^, throwing^49,77,79–83^, jumping^80^, hopping^64^, bench pressing^59^. Some studies described the CI effect in Pawlata roll learning^84^ and rifle shooting^85^. Non-sport skills included speech learning^53^ and laparoscopic skills^86,87^.

Other motor tasks included: the sequential key pressing task^88–91^, serial reaction time task^4,92,93^, sequential motor task^52,60,94^, visuomotor task^95^, motor sequence learning^96^, rapid aiming task^51,66,97^, pursuit tracking^98,99^, discrete sequential production task^61,100^ and time estimation task^101^.

#### Laboratory versus applied setting—comparison characteristics

Fifty-four studies were included in a laboratory vs. applied settings comparison. Five of these studies described results of immediate retention testing, of which two were conducted in laboratory settings^56,102^ and three in applied settings^46,103,104^. Thirty studies with reported results of delayed retention testing were carried out in the laboratory (89 effect sizes). In contrast, twenty-four studies describing delayed retention were conducted in applied settings (105 effect sizes).

Experiments conducted in laboratory settings included the following motor skills: sequential key pressing task^88–91^, sequential motor task^52,60,94^, serial reaction time task^4,92,93^, visuomotor task^95^, motor sequence learning^96^, rapid aiming task^51,66,97^, pursuit tracking^98,99^, oral motor learning task^63^ discrete sequential production task^61,100^ and time estimation task^101^.

In the study of Broadbent^75^, acquisition and retention of tennis skills were performed in laboratory settings. Learning and testing of golf skills and throwing were assessed by Chua et al.^49^. Similarly to Porter et al.^54^, participants practiced golf skills in the laboratory setting. In the study of Jeon^74^, the virtual reality-based balance tasks were performed using the Nintendo Wii Fit system. Moreno et al.^77^, in their study, focused on throwing in the laboratory setting—side throwing, low throwing, and darts throwing skills were learned and tested. Throwing and kicking skills presented in the experiment of Pollatou et al.^79^ were performed on the two apparatuses specially invented and constructed to measure the selected motor skills.

The study of speech training and testing^53^ by Wong et al. took place in the laboratory settings. The age of the participants in all the aforementioned laboratory experiments ranged from 11 years^75^ to 82 years^74^.

Studies conducted in applied settings were performed in natural environments (game-based or physical education class), examining: volleyball skills^57,69–71^, golf skills^47,58,67,68^, hockey^72,73^, soccer^65^, throwing skills^80–83^, basketball^55,62^, bench-pressing^59^, distance jumping^80^ and hopping^64^, as well as dancing skills^78^. Riffle shooting motor skills^85^ acquisition and testing were performed in indoor laboratories; however, all settings, including the position target height, followed the *Olympic and International Shooting Sport Federation* standards; therefore results of this experiment were included in the *applied setting* comparison.

Laparoscopic skills acquisition and testing were performed on medical students and post-graduate residents using a virtual reality simulator, mimicking the regular laparoscopic tasks^87^, using FLS Box trainer, in accordance to the *Fundamentals of Laparoscopic Surgery* (FLS) program^86^. The experiment utilizing Pawlata roll skill^105^ took place in the indoor pool.

The age of the participants in the group of applied studies ranged from 6 years^83^ to 34 years^62^. Adult participants were primarily students. An article by Souza and colleagues ^106^ was the only study describing the retention of motor skills in older adults (65–80 years old) in an applied setting. The motor task performed in the study consisted of throwing a boccia ball to three targets. However, due to missing data, this study was excluded from meta-analytic analysis.

#### The CI effect in youth vs. adults vs. elderly adults—comparison characteristics

All participants included in the present review were from 6 years^83^ to 82 years old^74^. Analyzed age subgroups were: young (up to 18 years old), adults (18 years old to 59 years old), and older adults (60 years and older). The articles covering immediate retention reported results from 68 children and 90 adults. In the delayed retention studies, 418 young participants, 205 older participants, and 1425 adults were included in further analyses. In the article by Tsutsui^82^, the authors presented results of 20 participants from 15 to 22 years old; therefore, the results of this study were not included in the age subgroups analyses.

#### The CI effect in novice vs. experienced participants—comparison characteristics

In his meta-analytic study, Brady initially compared the CI effect between skilled and novice participants. He classified their skill levels based on how the studies’ authors labeled them^7^. We applied the same rule in our review. Consequently, we classified participants of five studies as *skilled* (*n* = 202). Participants of these studies were characterized as follows.

In an article by Porter and Saemi, the skill level of participants was characterized in the following way: “participants (…) were considered moderately skilled at passing a basketball, which involved skills they were taught in their respective basketball course. None of the participants played college or professional basketball; however, some participants acknowledged that they did play basketball recreationally from time to time”^55^ (p. 64–65). Participants in the experiment on CI in learning throwing skills by Tsutsui and colleagues^82^ were labeled as *high-level pitchers* (*n* = 10) or *low-level pitchers* (*n* = 10). They were assigned to the aforementioned groups based on pretest scores. In their experiment, Broadbent and colleagues^107^ described the CI effect on young participants' learning of tennis skills. Based on their skill level, athletes were classified as *intermediate.* Years of participants’ experience in tennis ranged from 5.3 ± 2.2 in the blocked group to 5.9 ± 3.1 in the random group. In their study, Frömer and colleagues^76^ investigated the CI effect in learning virtual darts throwing. Based on pretest scores, it was apparent that the participants were familiarized with throwing but were not experts. Participants in the study of Porter and colleagues were classified as unskilled: “(…) had less than two years’ basketball playing experience (1.1 ± 1.3 years) and no representative level basketball playing experience”^62^(p. 7).

Summarizing, the aforementioned studies described different standards of inclusion to *skilled* group. Due to this fact, our meta-analytic comparison of *skilled* versus *novice participants* was not considered.

### Meta-analysis: results

#### Three-level mixed model

**Figure 2:**
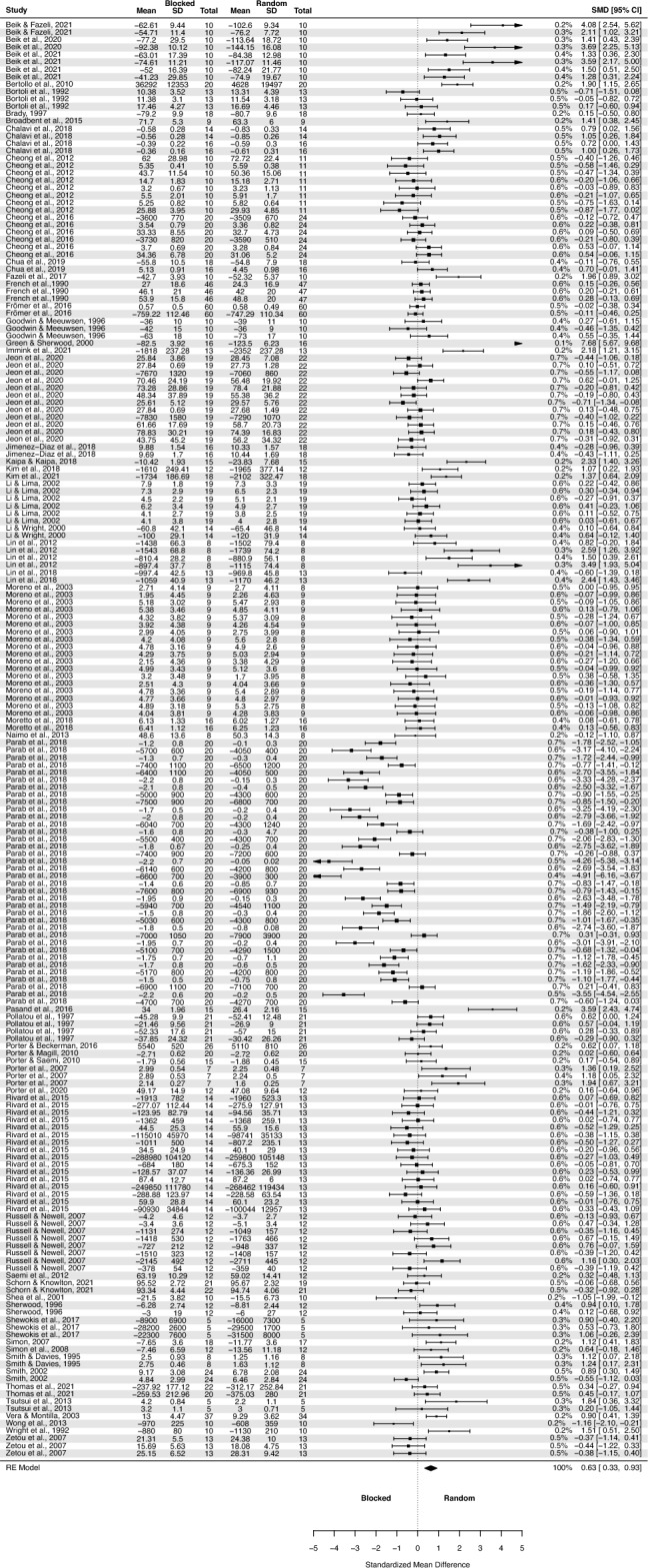
The three-level mixed model analysis of retention test results of random vs. blocked practice. The forest plot presents the results obtained by participants aged 6–82, including various motor tasks and different outcome measures. (studies descriptions in Appendix 3 https://osf.io/r59zs/?view_only=61397e4508384d13960936a556890962).

The analysis of contextual interference effect on delayed retention (Fig. 2) covered 54 primary studies and included 2068 participants, yielding 194 effect sizes (descriptions of the studies in Appendix 3 https://osf.io/r59zs/?view_only=61397e4508384d13960936a556890962 ).

The pooled effect size based on the three-level meta-analytic model was medium SMD = 0.63 (95% CI: 0.33, 0.93; p < 0.001). The estimated variance components (tau squared) were τ_3_^2^ = 0.93 and τ_2_^2^ = 0.34 for the level 3 and level 2 components, respectively. This means that *I*_3_^2^ = 66% of the total variation can be attributed to between-cluster, and *I*_2_^2^ = 24% to within-cluster heterogeneity. Total *I*^2^ = 90%.

Sensitivity analysis revealed that there were ten outcomes which were further than 4/n threshold: one outcome from Beik and Fazeli^94^; one from Beik et al.^52^; one from Bertollo et al.^78^; one from Green and Sherwood^66^; one from Immink et al.^96^; one from Kaipa and Kaipa^63^; one from Lin et al.^92^; one from Pasand et al.^70^; one from Shea et al.^48^; and from Wong et al.^53^.

After having removed the outliers, the pooled effect size was small SMD = 0.43 (95% CI: 0.19, 0.67; p < 0.001). The estimated variance components (tau squared) were τ_3_^2^ = 0.44 and τ_2_^2^ = 0.28; *I*_3_^2^ = 51% and *I*_2_^2^ = 33%; respectively. The outliers had a substantial effect on pooled effect size, i.e. SMD decreased from 0.63 (with outliers included) to 0.43 (without outliers).

Given that 36 outcomes were retrieved from the Parab’s et al. study^64^ we also performed a sensitivity analysis removing all 36 outcomes. The result was not substantially different from the full analysis: SMD = 0.69 (95% CI: 0.41, 0.97; p < 0.0001).

#### Random-effects model with averaged SMDs

**Figure 3:**
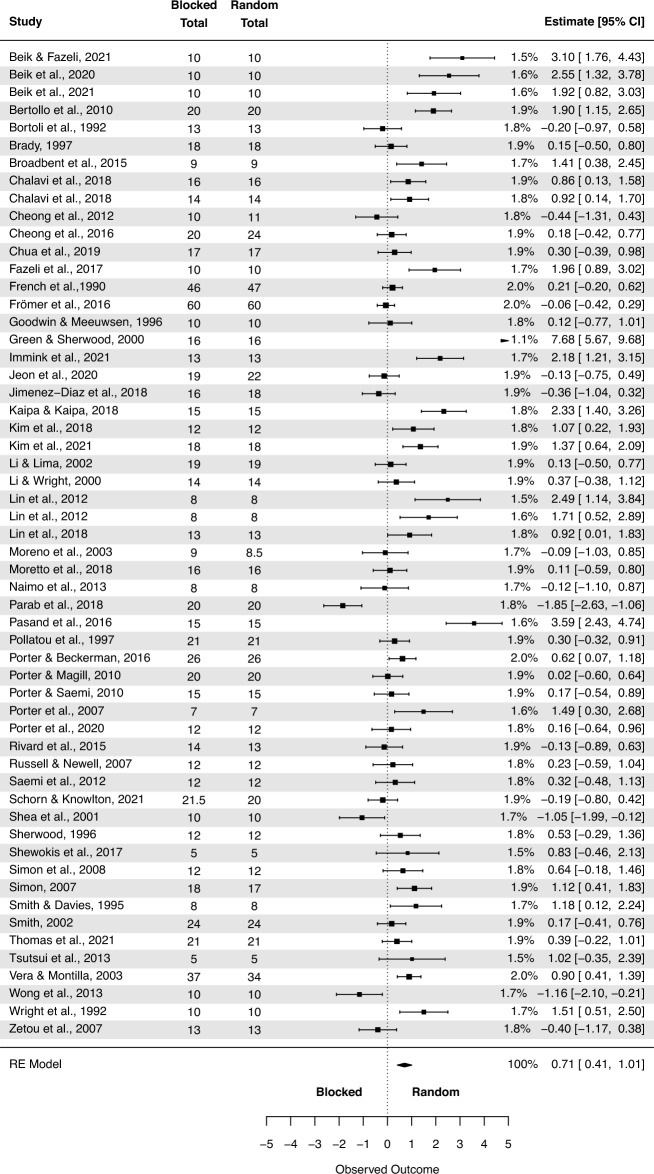
The random-effects model analysis of retention test results of random vs. blocked practice. In columns “Random Total” and “Blocked Total” are provided number of participants.

The analysis based on averaged SMDs (form one study) yielded the following: SMD = 0.71 (95% CI: 0.41, 1.01; p < 0.001) (Fig. 3). The estimated variance components (tau squared) were τ^2^ = 1.3. Total *I*^2^ = 88.5%. The sensitivity analysis without two outcomes^66,70^ which were further than 4/n threshold, yielded the following SMD = 0.56 (95% CI: 0.32, 0.80; p < 0.001), τ^2^ = 0.64. Total *I*^2^ = 81.69%.

#### Laboratory vs. field-based (applied) studies

The included studies were divided into those carried out in a laboratory setting (n = 30), including 1210 participants (89 effect sizes), and the remaining (n = 24) conducted in an applied setting (105 effect sizes), including 858 participants.

##### Three-level mixed model

**Figure 4:**
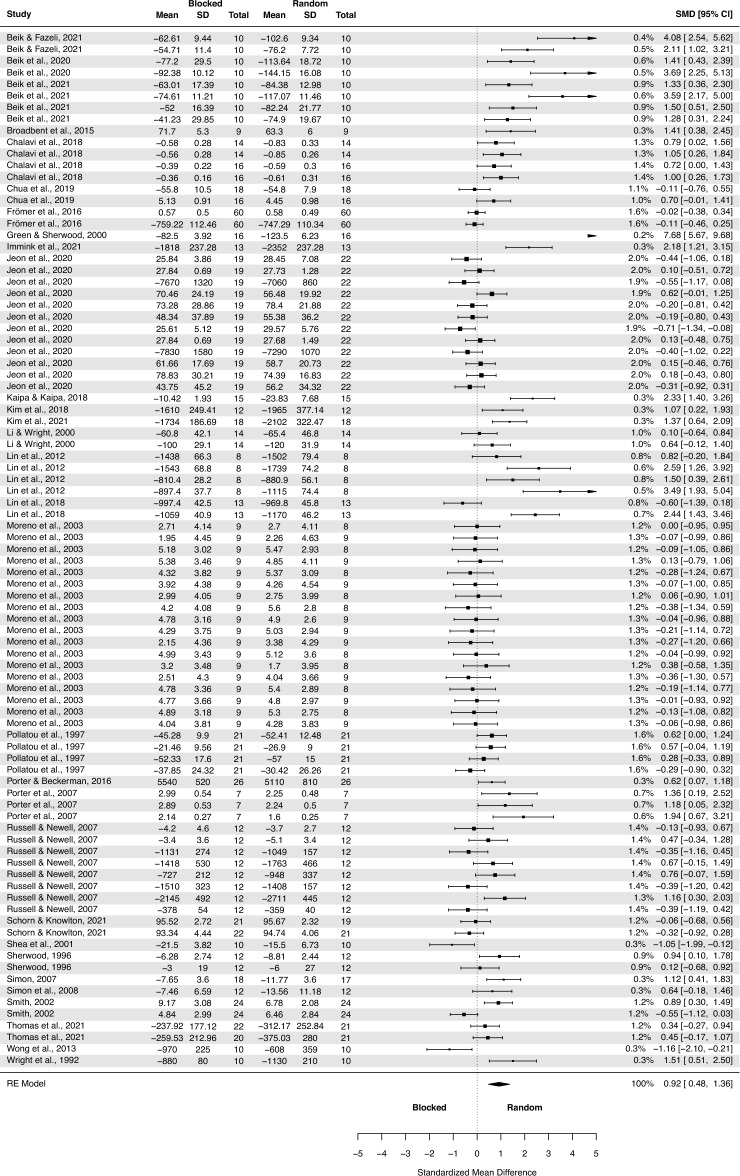
The three-level mixed model analysis of retention test results in a random and blocked schedule in a laboratory setting. The forest plot presents the retention test results obtained by participants practicing in a laboratory setting, including various motor tasks and different outcome measures.

A subgroup analysis of the CI effect in laboratory studies was performed (Fig. 4). The test of moderators turned out to be significant F(1, 192) = 4.50, p = 0.03.

The pooled effect size based on the three-level meta-analytic model was large SMD = 0.92 (95% CI: 0.48, 1.36; p < 0.001). The estimated variance components (tau squared) were τ_3_^2^ = 1.28 and τ_2_^2^ = 0.11 for the level 3 and level 2 components, respectively. Heterogeneity was high, *I*_3_^2^ = 83% and *I*_2_^2^ = 7%; total *I*^2^ = 90%. Sensitivity analysis revealed that after removing four outcomes, i.e. one from Green and Sherwood^66^; one from Lin et al.^92^; one from Shea et al.^48^; and one from Wong et al.^53^, the pooled effect size increased SMD = 0.93 (95% CI: 0.59, 1.23; p < 0.001).

**Figure 5:**
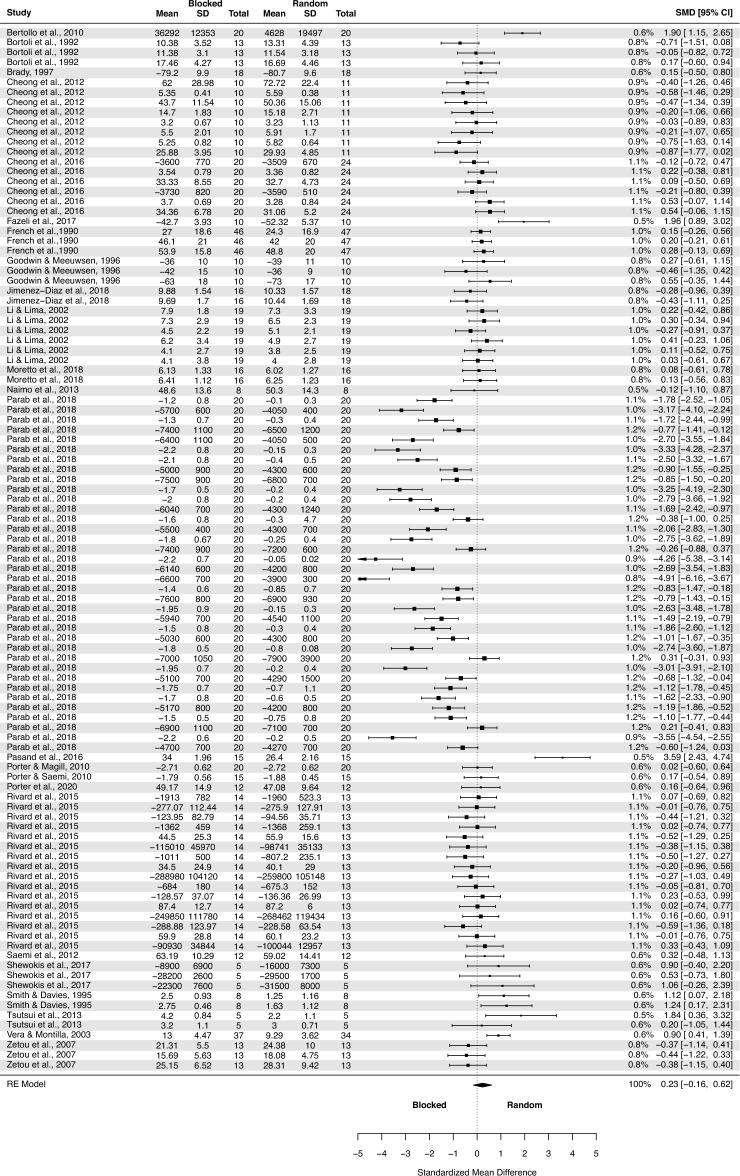
The three-level mixed model analysis of random and blocked schedule retention test results in an applied setting. The forest plot presents the retention test results in an applied setting, including various motor tasks and different outcome measures.

Analogously, a subgroup analysis of the CI effect in applied studies was conducted (Fig. 5). The non-significant pooled effect size based on the three-level meta-analytic model was small SMD = 0.23 (95% CI: -0.16, 0.62; p = 0.24). The estimated variance components (tau squared) were τ_3_^2^ = 0.58 and τ_2_^2^ = 0.48 for the level 3 and level 2 components, respectively. Heterogeneity was high, *I*_3_^2^ = 48% and *I*_2_^2^ = 40%; total *I*^*2*^ = 89%.

Sensitivity analysis revealed that after removing three outcomes, i.e. one from Bertollo et al.^78^; one from Fazeli et al.^68^, and one from Pasand et al.^70^, the pooled effect size decreased to negligible and favoring blocked practice SMD = −0.01 (95% CI: −0.35, 0.32; p = 0.94).

##### Random-effects model with averaged SMDs

The test of moderators was statistically significant QM(1) = 4.11, p = 0.04.

**Figure 6:**
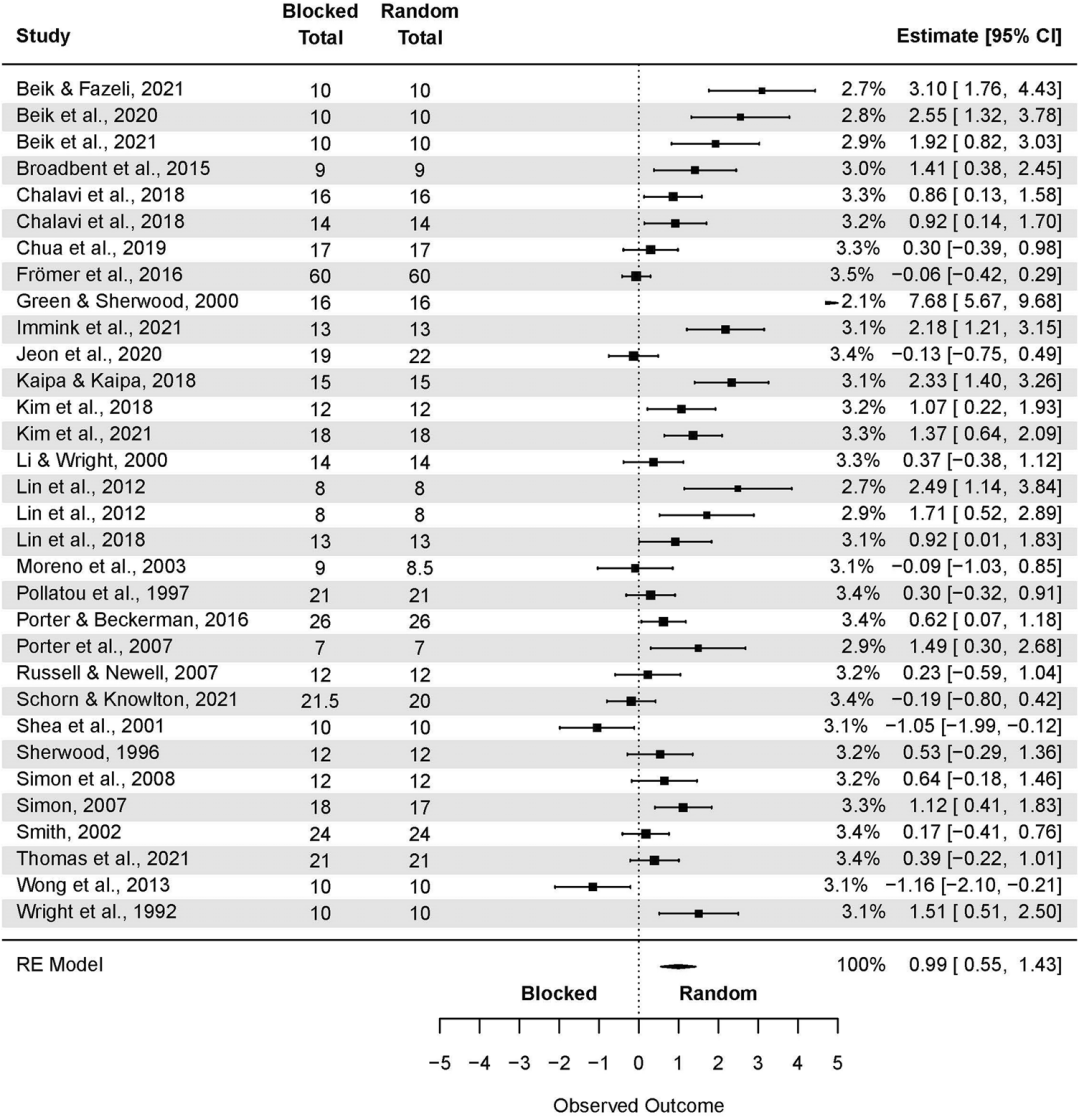
The random-effects model analysis of retention test results of random vs. blocked in an applied setting. In columns “Random Total” and “Blocked Total” are provided number of participants.

For the studies in laboratory settings (Fig. 6), the model yielded the following: SMD = 0.99 (95% CI: 0.55, 1.43; p < 0.001), τ^2^ = 1.39. Total *I*^*2*^ = 81.69%. Green and Sherwood’^66^ results were removed during sensitivity analysis, yielding the following: SMD = 0.82 (95% CI: 0.49, 1.16; p < 0.001); τ^2^ = 0.70; *I*^2^ = 82.35%.

**Figure 7:**
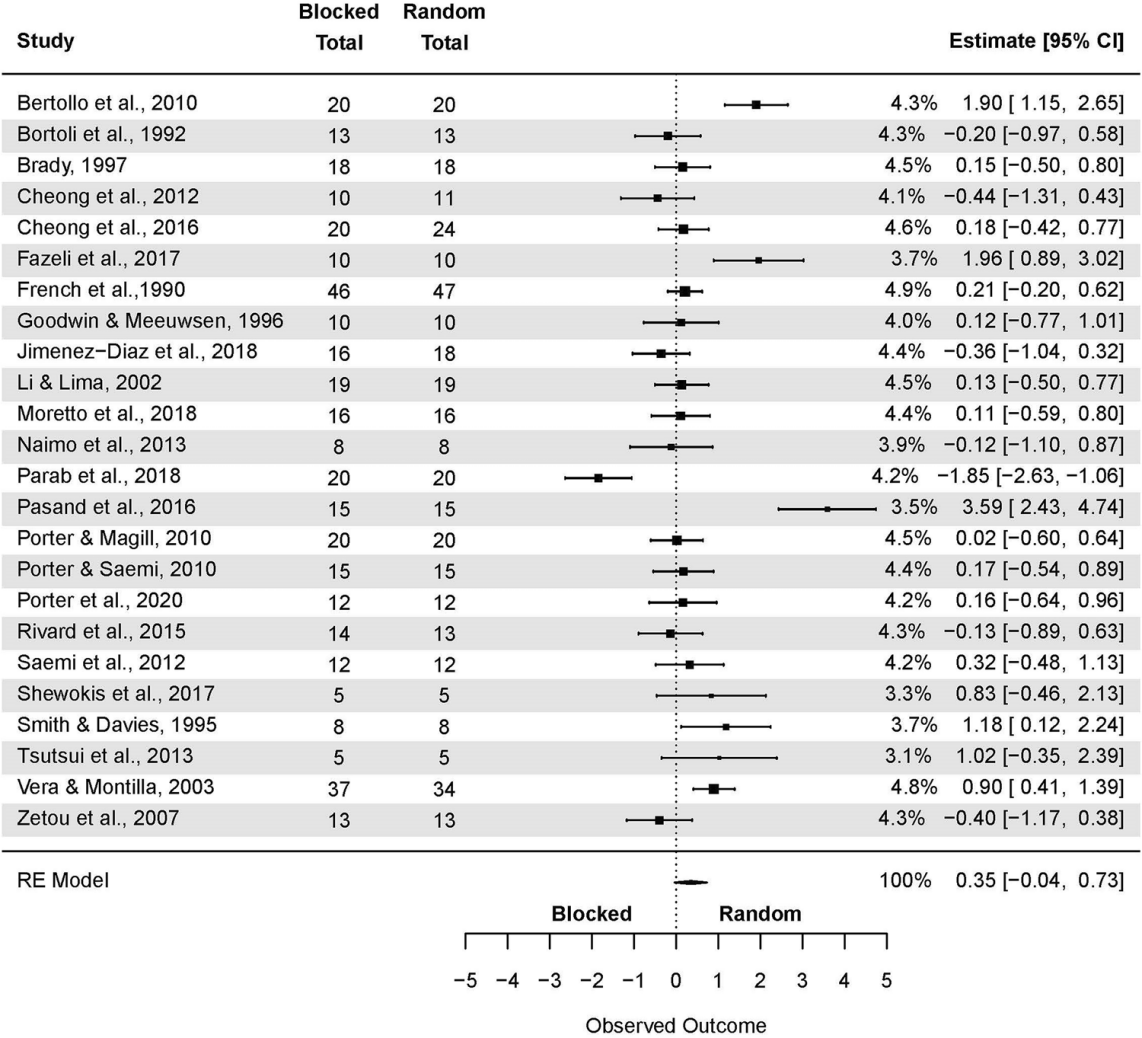
The random-effects model analysis of retention test results of random vs. blocked in a laboratory setting. In columns “Random Total” and “Blocked Total” are provided number of participants.

The model yielded the following for the studies in applied settings (Fig. 7): SMD = 0.35 (95% CI: −0.04, 0.73; p 0.08), τ^2^ = 0.75. Total *I*^2^ = 84.33%. Two outcomes were removed during sensitivity analysis, i.e. Parab et al.^64^ and Pasand et al.^70^. The model changed to SMD = 0.30 (95% CI: 0.04, 0.56; p = 0.02); τ^2^ = 0.22; *I*^2^ = 61.85%.

#### The CI effect in young vs. adults vs. elderly adults

Fifty-three studies were included in a meta-analytic comparison of the CI effect in three age groups, resulting in the testing of 205 older adults in total, 1425 adults, and 418 young participants. Participants (aged from 15 to 22 years old) from the study of Tsutsui^82^ were excluded from this comparison due to difficulty in qualifying them to any of the abovementioned age groups. This analysis yielded 6169 measurements in total and elicited: 49 effect sizes for young participants, 119 effect sizes for adults, and 24 effect sizes for the group of older adults.

##### Three-level mixed model

The test of moderators was not significant F(2, 189) = 1.69, p = 0.19; however we decided to perform the analysis anyway, because the differences between age groups (SMD) were quite substantial.

**Figure 8:**
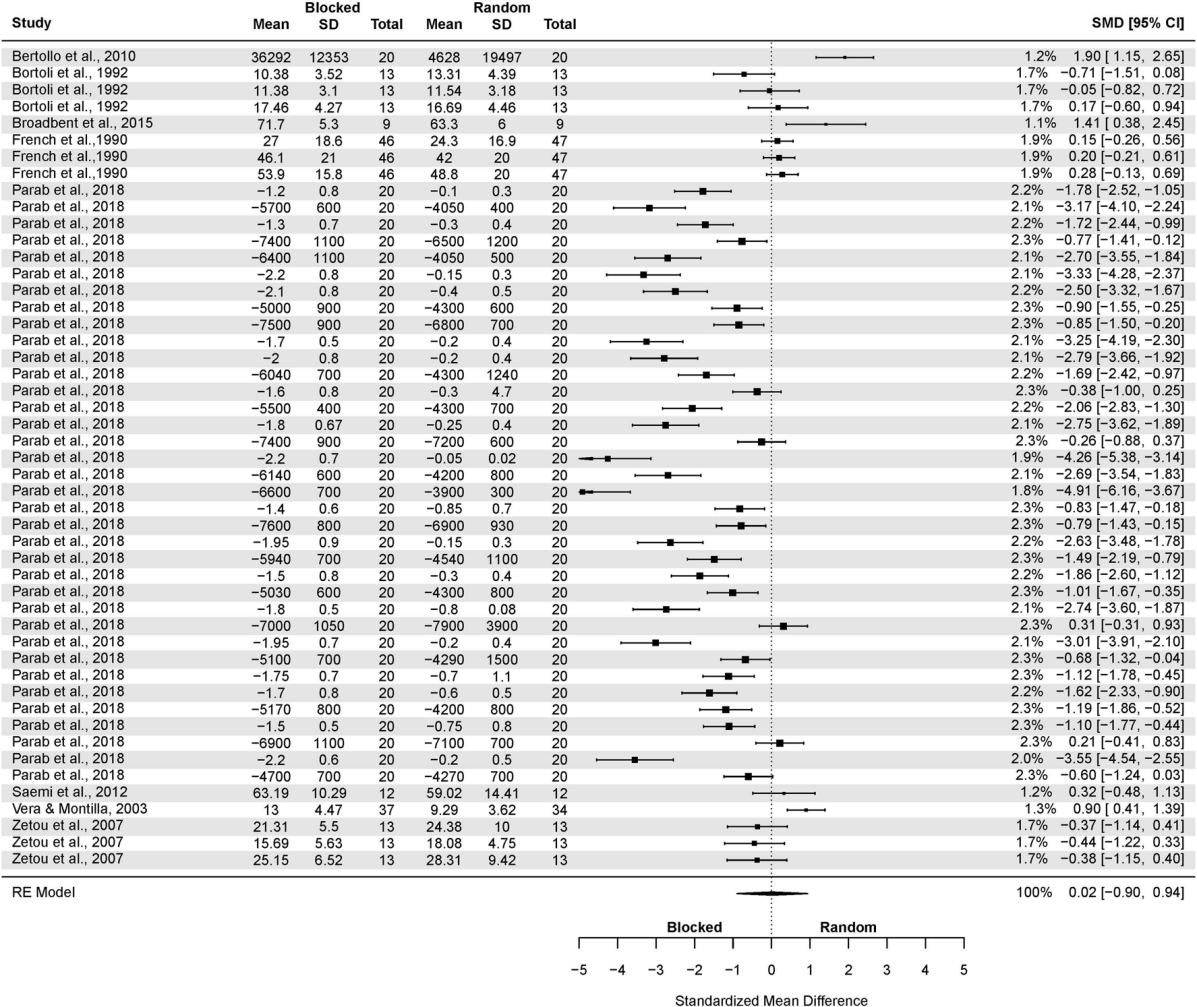
The three-level mixed model analysis of young participants’ retention tests results: random practice vs. blocked practice. The forest plot presents the retention test results obtained by participants aged 6–18, including a variety of motor tasks and different outcome measures.

The pooled effect size based on the three-level meta-analytic model for the subgroups of young participants was negligible SMD = 0.02 (95% CI: -0.90, 0.94; p = 0.97). The estimated variance components (tau squared) were τ_3_^2^ = 1.04 and τ_2_^2^ = 1.05 for the level 3 and level 2 components, respectively. Heterogeneity was high, *I*_3_^2^ = 47% and *I*_2_^2^ = 47%; total *I*^2^ = 94%. (Fig. 8). Sensitivity analysis revealed that after removing two outcomes, i.e. one from Bertollo et al.^78^; and one outcome from Broadbent et al.^75^ the pooled effect size decreased to small SMD = −0.39 (95% CI: −1.30, 0.51; p = 0.39).

**Figure 9:**
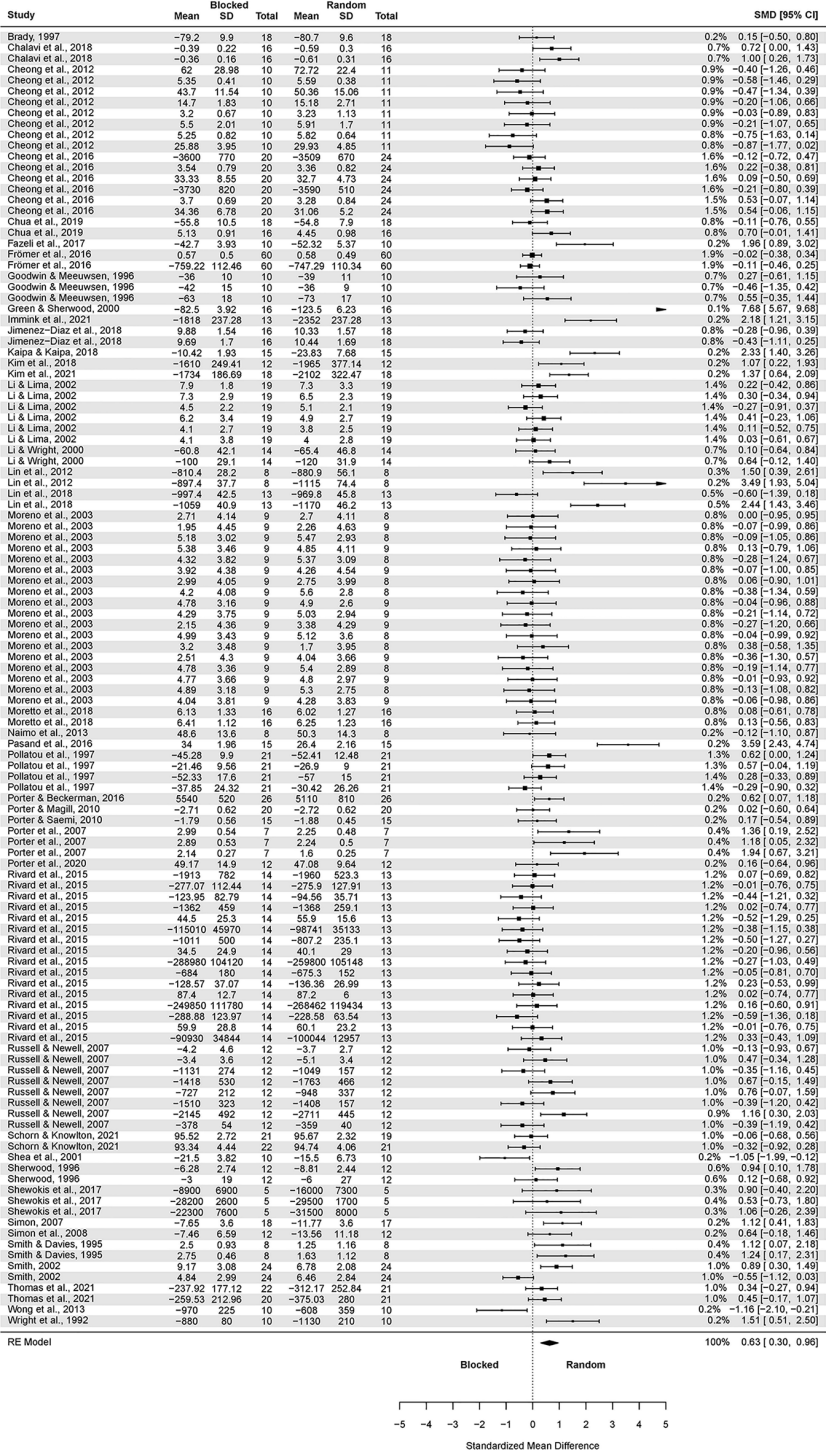
The three-level mixed model analysis of adult participants’ retention tests results: random practice vs. blocked practice. The forest plot presents the retention test results obtained by participants aged 18–59, including various motor tasks and different outcome measures.

The adult’s pooled effect size was medium SMD = 0.63 (95% CI: 0.30, 0.96; p = 0.54) (Fig. 9). The estimated variance components (tau squared) were τ32 = 0.42 and τ22 = 0.53 for the level 3 and level 2 components, respectively. Heterogeneity was high, I23 = 39% and I22 = 48%; total I2 = 86.82%. The estimated variance components (tau squared) were τ_3_^2^ = 0.99 and τ_2_^2^ = 0.03 for the level 3 and level 2 components, respectively. Heterogeneity was *I*_3_^2^ = 85% and *I*_2_^2^ = 2%; total *I*^2^ = 87%.

Sensitivity analysis revealed that after removing seven outcomes, i.e. Green and Sherwood^66^; one from Immink et al.^96^; one from Kaipa and Kaipa^63^; one from Lin et al.^92^; one from Pasand et al.^70^; one from Shea et al.^48^; and from Wong et al.^53^, the pooled effect size decreased to small SMD = 0.45 (95% CI: 0.24, 0.66; p < 0.001).

**Figure 10:**
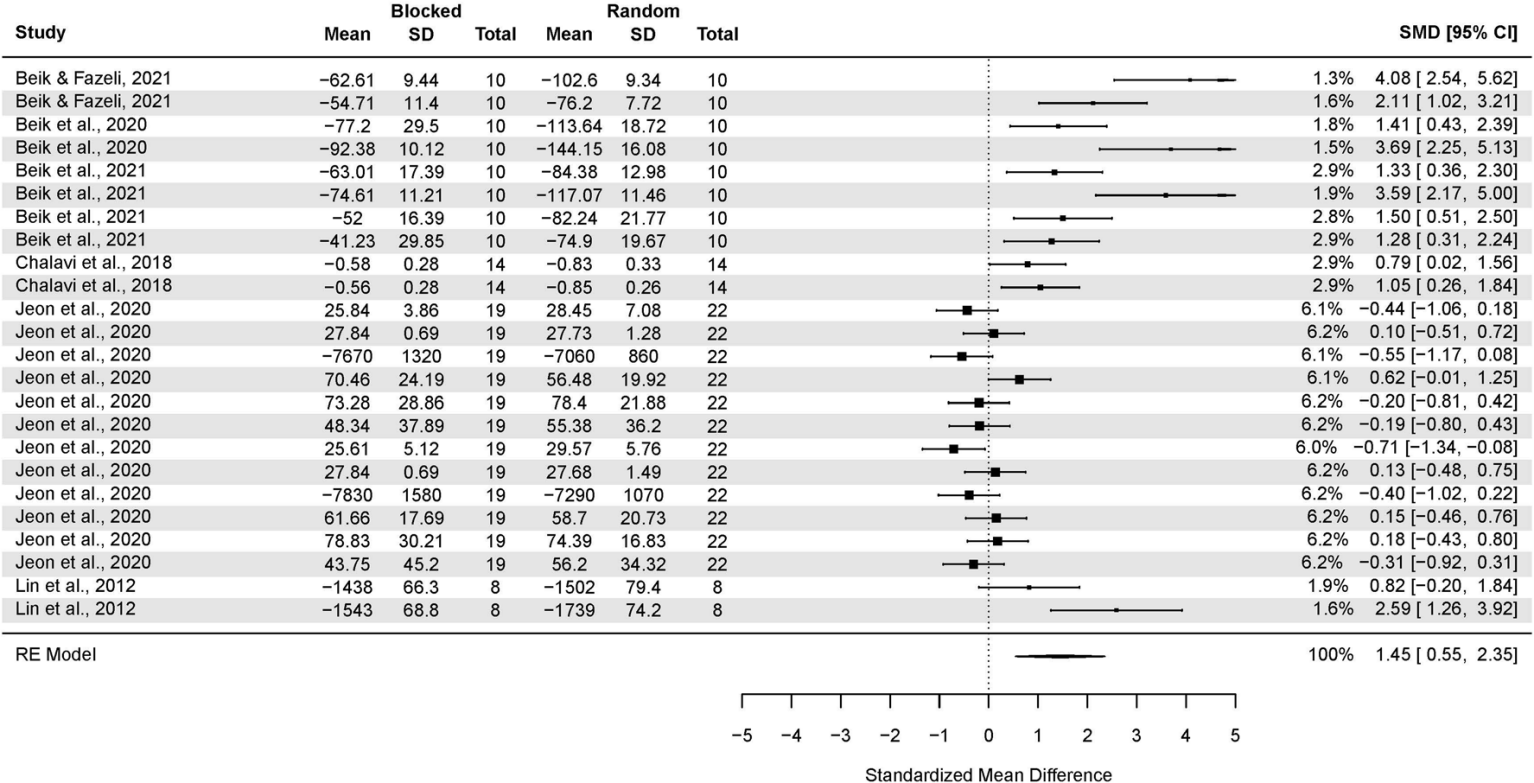
The three-level mixed model analysis of older adults’ retention tests results: random practice vs. blocked practice. The forest plot presents the retention test results obtained by participants aged 60–82, including a variety of motor tasks and different outcome measures.

The pooled effect size for older adults was large SMD = 1.45 (95% CI: 0.55, 2.35; p = 0.003) (Fig. 10). The estimated variance components (tau squared) were τ_3_^2^ = 0.99 and τ_2_^2^ = 0.10 for the level 3 and level 2 components, respectively. Heterogeneity was high, *I*^2^_3_ = 80% and *I*^2^_2_ = 8%; total *I*^2^ = 88%.

In sensitivity analysis, one outcome from Beik et al.^52^ was removed yielding large pooled effect size SMD = 1.64 (95% CI: 0.53, 2.75; p = 0.005).

##### Random-effects model with averaged SMDs

Test of Moderators turned out to be insignificant: QM(df = 2) = 4.50, p = 0.11.

**Figure 11:**
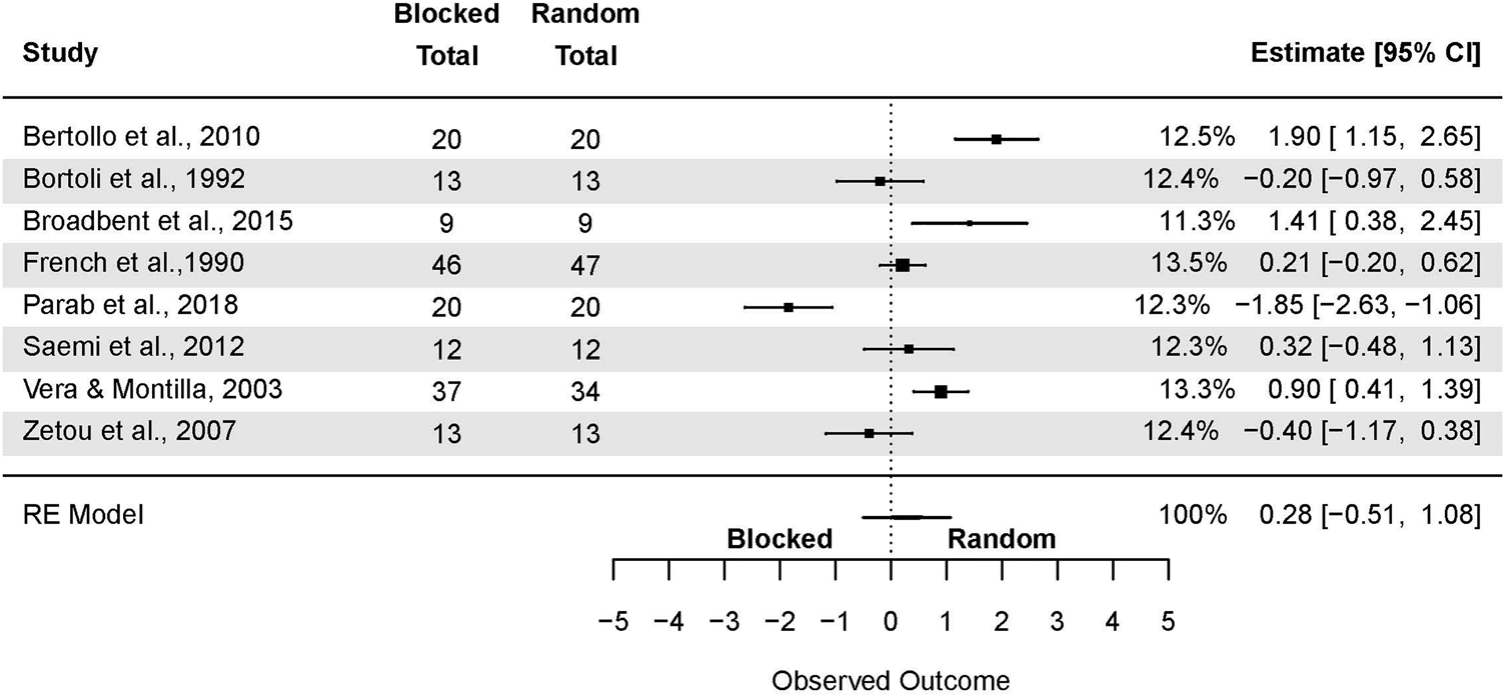
The random-effects model analysis of young participants’ retention tests results: random practice vs. blocked practice. In columns “Random Total” and “Blocked Total” are provided number of participants.

For the studies with young participants, the model yielded the following: SMD = 0.28 (95% CI: -0.51, 1.08; p = 0.48); τ^2^ = 1.17; *I*^*2*^ = 91.07% (Fig. 11). During sensitive analysis, Parab’s et al.^64^ outcomes were removed and, as a result, SMD = 0.58 (95% CI: −0.03, 1.18; p = 0.06); τ^2^ = 0.53; *I*^2^ = 82.83%.

**Figure 12:**
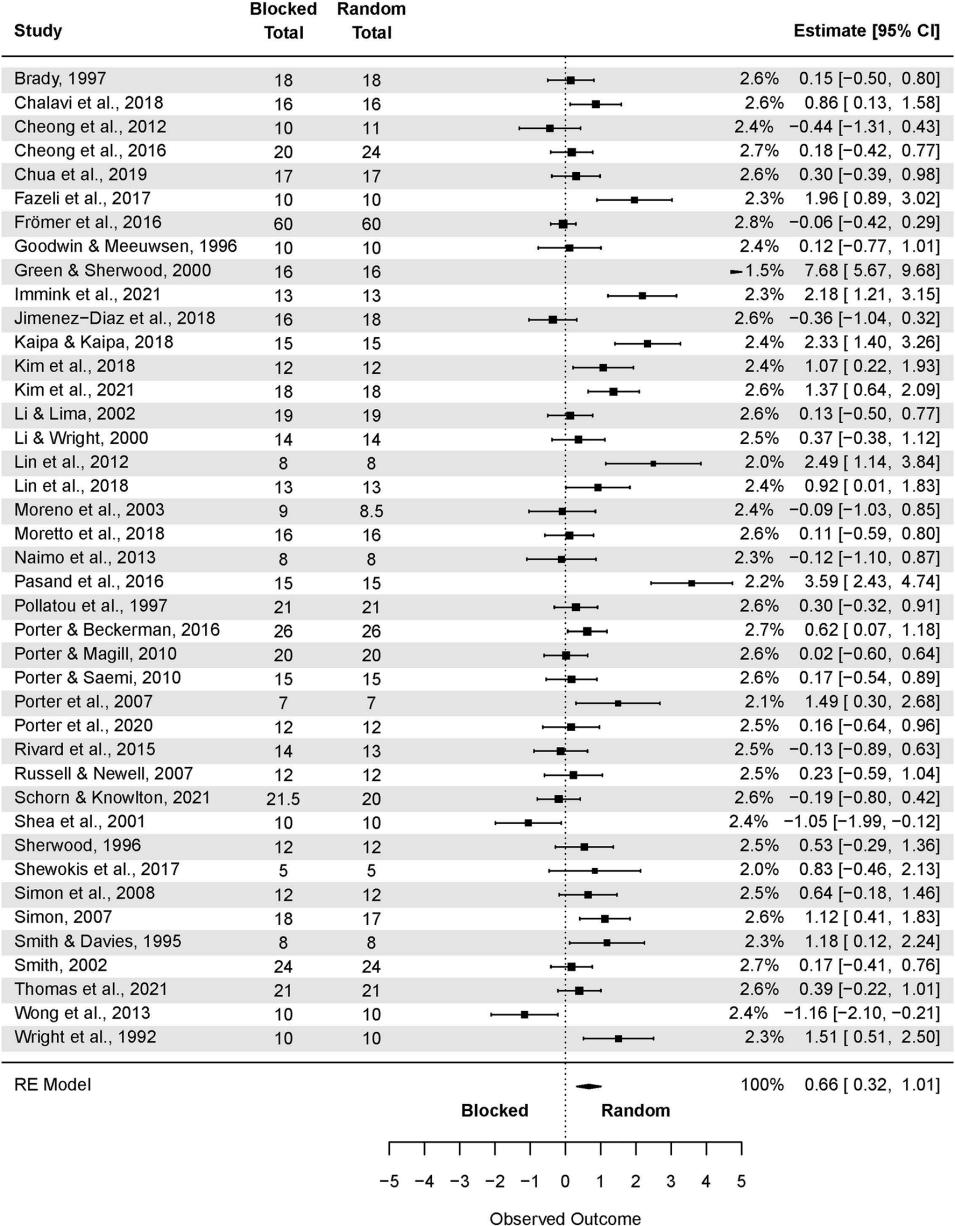
The random-effects model analysis of adults’ participants’ retention tests results: random practice vs. blocked practice. In columns “Random Total” and “Blocked Total” are provided number of participants.

For the studies with adults, the model yielded the following: SMD = 0.66 (95% CI: 0.32, 1.01; p < 0.001), τ^2^ = 1.06; *I*^2^ = 87.84% (Fig. 12). Two studies were removed based on sensitivity analysis, Green and Sherwood^66^ and Pasand et al.^70^. The model yielded the following SMD = 0.47 (95% CI: 0.23, 0.70; p = 0.06); τ^2^ = 0.39; *I*^2^ = 73.22%.

**Figure 13:**
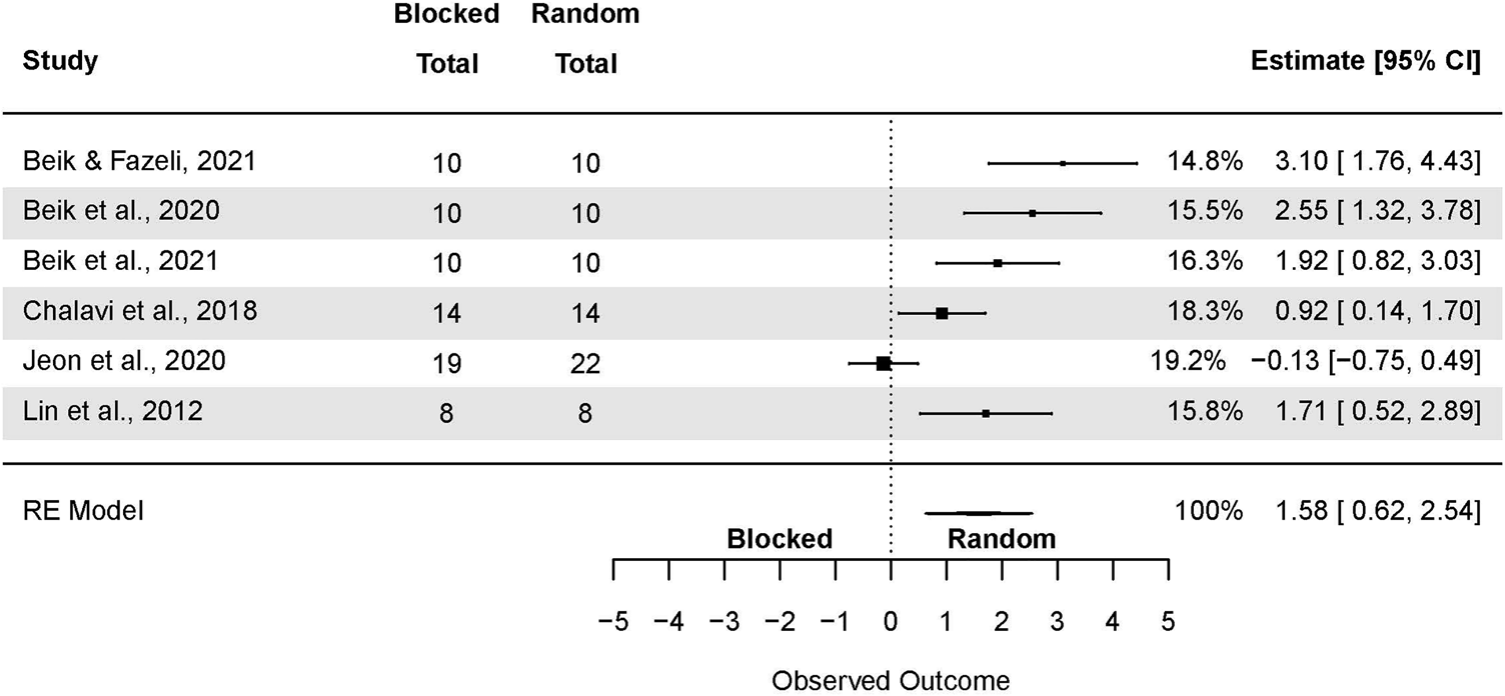
The random-effects model analysis of older adult’s participants’ retention tests results: random practice vs. blocked practice. In columns “Random Total” and “Blocked Total” are provided number of participants.

For the studies with older adults, the model yielded the following: SMD = 1.58 (95% CI: 0.62, 2.54; p = 0.001), τ^2^ = 1.15; *I*^2^ = 82.63% (Fig. 13). There were no outliers further than 4/n distances from the threshold.

#### Risk of publication bias assessment

Substantial heterogeneity (high *I*^2^ values) was present in almost every analysis. According to Van Aert and colleagues, none of the publication bias methods has desirable statistical properties under extreme heterogeneity in true effect size^108^. Therefore, plotting a funnel plot as a visual aid for detecting bias or systematic heterogeneity, in this case, was doubtful. We did not apply other analysis methods, i.e., Egger’s regression test or rank-correlation test.

The possible reasons for high *I*^*2*^ values are elaborated on in the “Discussion”*.*

## Discussion

The study's main objective was to determine the effect size of CI on retention in motor learning. When applying the three-level mixed model, we found that the pooled effect size was statistically significant and medium (SMD = 0.63). Analysis with the random-effects model on averaged outcomes for singles studies yielded similar results, i.e., statistically significant medium effect (SMD = 0.71).

Our secondary objectives were to estimate the CI effect in laboratory versus non-laboratory studies and estimate the CI effect in different age groups. Only analysis of laboratory studies yielded statistically significant results, and the SMD was large (the three-level mixed model SMD = 0.92; the random-effects model SMD = 0.99). Analysis of applied studies turned out to be statistically insignificant, and the effect size was slightly above negligible (SMD = 0.23 in the three-level mixed model and SMD = 0.28 in the random-effects model). In both analyses, random practice was favored.

Lee and White^109^ suggested that CI effect is more conspicuous in laboratory settings as tasks are less motor demanding, more cognitively loaded, lack intrinsic interest, and quickly reach an asymptote. The robustness of the CI effect in a laboratory setting, on the other hand, might be attributed to its well-controlled specification. Additionally, as Jeon and colleagues^74^ noticed, the CI effect in laboratory settings was frequently associated with simple tasks. In contrast, CI in more complex tasks was often examined in the field setting. One plausible explanation of this finding may be that the complexity of the sport task, alongside high interference practice, could be too challenging for the information processing system, negatively affecting learning^110,111^. Therefore, the CI effect in applied setting may not be so conspicuous.

The analyses in age groups yielded significant results in older adults in both analyses. In the first place, CI effect in motor learning of older adults was large (in the three-level mixed model SMD = 1.45 and SMD = 1.58 in the random-effects model), i.e., a random schedule was more beneficial for retention.

The benefits of implementing random practice in motor learning for adult participants were medium (SMD = 0.63 in the three-level mixed model and SMD = 0.66 in the random-effect model). However, the difference between the blocked and random groups of adults was insignificant in the three-level mixed model, whereas it was significant in the random-effect model. The CI effect in young participants was statistically insignificant, and the SMD was negligible in the three-level model (SMD = 0.02) and small in the random-effects model (SMD = 0.28).

The overall results of our review partially corresponded with those reported in the meta-analysis by Brady^7^. In line with the constantly advancing methodology of conducting meta-analyses, the inclusion criteria implemented in this review were more thoroughly detailed than those presented in Brady’s study^7^. Consequently, 13 following studies included in Brady’s meta-analysis were considered irrelevant in the present review and, therefore, excluded. The primary studies by Landin and Hebert^112^, Sekiya and colleagues^113–115^, Wulf and Lee^116^ described serial practice order instead of random schedule. In the studies by Hebert^110^, Smith^117^, Smith and colleagues^118^, Wrisberg and Liu^119^ alternating practice instead of random schedule was presented. In the article by Hall and Magill^120^, experiment described by Lee and colleagues^121^, and a study by Shea and Titzer^122^ multiple task learning was implemented. In the article by Bortoli and colleagues^123^, included in Brady’s meta-analytic study^7^, constant and variable practice schedules were compared. Unfortunately, data from 19 primary studies, included in his meta-analysis, were not available. Therefore, 12 primary articles included in Brady’s study^7^ constituted to our meta-analysis, yielding 29 effect sizes.

The remaining 42 primary articles included in our study (yielding 165 effect sizes) were not included in Brady’s study^7^. Despite this fact, the outcomes of the present review supported previous results of the CI effect described by Brady. In his study, the effect size of the randomized schedule in a laboratory setting was medium (ES = 0.57)—similarly to the outcome obtained in the present meta-analysis. Furthermore, Brady^7^ reported a different magnitude of randomization for adults and young participants. The CI effect in motor learning of young participants (ES = 0.10) was described as *almost trivial*, whereas the randomization effect in motor learning of adults was considered *roughly moderate* (ES = 0.35). Our findings were consistent, showing significant differences of CI effect between age groups. The pooled effect size for young participants was also negligible, the beneficial effect of randomization in adults was medium. The effect size of CI effect in the group of older adults was close to large and this age group is hard to compare since Brady did not recognized older adults as a separate subgroup.

Our results are different from those reported by Ammar and colleagues^18^. We found that the pooled effect size was medium 0.63 while Ammar et al. reported small. Probably the differences we found may be attributed to the search strategies, number of studies and effects sizes included in both meta-analysis. Ammar et al. omitted EBSCO database (including APA PsycArticles, APA PsychInfo, SPORTDiscus with Full Text, Medline, and Academic Search Complete), searching a publisher database instead (Taylor and Francis). They finally included 28 studies and 84 ES whereas in our meta-analysis 54 studies and 194 ES were included. Similarly, the differences in subgroups analysis can be explained in methods applied.

### Age- and settings-related differences

The most interesting trend found in our and Brady’s^7^ meta-analysis was that the CI effect is more conspicuous in older adults. However, what has to be emphasized is that all included experiments with older adults' participation were performed in a laboratory setting. On the opposite, only one primary study^75^, including 18 young participants, was conducted in a laboratory setting. The remaining studies, including 400 youth, were performed in an applied setting. Due to this fact, it is difficult to differentiate whether participants' age plays a crucial role in the CI effect. Perhaps it is the setting that is critical when considering the CI effect? To solve this problem, more studies on children in a laboratory and more studies on older adults and elderly in an applied setting could be conducted. Given the disparity between settings in different age groups, the overall CI effect in different age groups may be biased. Analogically, one could claim that the results of the settings comparison may be biased, i.e., in the applied setting, only children (except for one study) and no older adults are included. The opposite situation is noticed in a laboratory setting.

Ammar and colleagues^18^ reported that CI effect was present only in 20–24 and 25–32-year-old participants (small and moderate ES, respectively) whereas blocked practice was favored in older adults. Again, these contradictions to our findings may be attributed to the different search methods and the number of studies and effect sizes included in both meta-analysis.

### Low quality and bias problem

Given the results of the methodological assessment of the included studies, we need to consider that the CI effect as described here may be biased. According to the Quality Assessment Tool for Quantitative Studies, only three articles out of 54 presented moderate or high quality^26^. None of the study’s protocols was published prior to the study's commencement.

Participants were not likely to be representative in 37 out of 59 studies included in this review. Additionally, the answer was “Can’t tell” in ten other studies. In 47 out of 59 studies, the sample was not representative of the target population. In 38 out of 59 studies, the question about the critical differences between groups prior to the intervention was answered: “Can’t tell”. In 56 out of 59 studies, there was no information on whether the assessor(s) was aware of the intervention or the exposure status of participants. In 39 out of 59 studies, there was no information on whether participants were aware of the study research question. 51 out of 59 studies did not report the withdrawal and dropout numbers and reasons. Unfortunately, studies on the CI effect are of poor quality. When considering all of the limitations of the included studies, we cannot tell whether participants were aware of the study research question. One may conclude that studies on CI may be biased. Therefore, the question initially asked by Al-Mustafa^11^ and re-asked by Brady^7^ has to be re-stated again: is "contextual interference a laboratory artifact or sport-skill related''?

### Heterogeneity problem

The possibility of substantial heterogeneity in the analyses was considered when planning our review and introducing the broad PICO criteria. Differences in populations such as age and origin, followed by a variety of included motor tasks and outcome measures, could contribute to increased heterogeneity. Additionally, the number of primary studies (and consequently different methodologies such as experiment duration) could determine the level of heterogeneity. According to Van Aert and colleagues: “*none of the publication bias methods has desirable statistical properties under extreme heterogeneity in true effect size”*^108^(p.8)*.* Another source of heterogeneity could be the low quality of the included studies.

There are many reasons why *I*^2^ values could have been so high. First of all, it may be due to the test we used. We used *I*^2^ index instead of *Q* test that was used by Brady (2004). It is because *Q* test informs about the presence versus the absence of heterogeneity, whereas *I*^2^ index also quantifies the magnitude of heterogeneity^124^. Second, in all our analyses, more than 24 outcomes were included. In the general analysis, 194 outcomes were used. However, the more studies are included in the heterogeneity tests the higher is *I*^2^ value^125^. As Schroll and colleagues^126^ noted, if there are very few studies included in the meta-analysis, with relatively few participants, the risk of high heterogeneity as quantified with *I*^2^ value (> 50%) is very small, although the heterogeneity may be present. Unfortunately, authors dealing with high heterogeneity cannot do much about it. Increased precision does not solve the problem^127^, and there is little advice for authors on how to deal with it^126^.

### Limitations

Firstly, there is a dependency within studies problem when multiple outcomes from a single study come from the same sample. Unfortunately, there is no simple answer to how to deal with such a problem. The most reliable approach would be to calculate the correlations between outcomes from the same sample using the raw data. This approach is, however, infeasible—many authors do not want to share their results, many have already lost them, and many authors passed away (given that we were analyzing results from 1966).

Additionally, the most popular approaches include averaging effect sizes derived on the same sample, analyzing each type of outcome separately, and applying three-level mixed models. Again, each of these approaches has its advantages and limitations. In our case, we decided that the most suitable would be to apply the three-level mixed model. Though the model assumes that there is no correlation between outcomes (effects sizes) obtained from the same sample, as Van den Noorgate et al.^41^ noticed, “An important conclusion is that, although the multilevel model we proposed for dealing with multiple outcomes within the same study in principle assumes no sampling covariation (or independent samples), our simulation study suggests that using an intermediate level of outcomes within studies succeeds in accounting for the sampling covariance in an accurate way, yielding appropriate standard errors and interval estimates for the effects” (p.589). Nevertheless, one may doubt the model since it assumes the effect sizes are independent.

To compare our results, we applied another model, the classical random-effects model; however, we used averaged effect sizes whenever they were derived from the same studies (same population). This approach is not potentially biased due to the dependency problem; however, it loses its informative value since the variance between effect sizes is reduced^41^.

What is worth mentioning is that both models we applied yielded very similar results.

One could suggest that we could analyze each outcome type, e.g., grouping them according to the skill characteristic (throws, aiming, kicking, etc.) or nature of the outcome (force production, scoring system, reaction time, movement time, kinematic characteristics, etc.). This approach would require a robust theoretical framework to differentiate different outcomes. Our potential readers should consider the limitations and advantages of our analyses.

Secondly, we did not study practice volume or nominal task difficulty in our analyses. These may be important factors contributing to the overall pooled effect size; however, given our review is broad in scope, more detailed analyses could be performed in the subsequent studies.

Thirdly, an analysis focusing exclusively on experience as a contributing factor affecting CI effect could be performed. However, a thorough and detailed definition of experience might be used.

### Recommendation for future research

There is a limited number of motor learning studies utilizing young (up to 18 years old) healthy participants in a laboratory setting. Most motor learning studies with older adults (60 years and older) are performed in a laboratory setting. Therefore, we recommend further research on the CI effect, including young participants in a basic (laboratory) setting. We would also suggest future research on the CI effect in older adults (60 years and older) conducted in an applied setting.

In the subsequent studies, researchers could put a strong emphasis on the quality (methodology) of the research.

## Conclusions

The Ci effect is a robust phenomenon in motor learning. Our results evinced, however, that, similarly to Brady (2004), this claim is primarily based on laboratory studies in adults and older adults. Experiments conducted in applied settings yielded fewer convincing results. Moreover, high CI does not benefit retention in young participants. It does in adults and older adults.

Practitioners, should consider other factors, e.g., the interaction between the skill level of the performer's motor complexity, cognitive load, and the performer’s intrinsic interest, while deciding how to structure practice and how much CI apply.

## Data Availability

Data is available in the open repository, https://osf.io/r59zs/?view_only=61397e4508384d13960936a556890962

## References

[1] Raviv L, Lupyan G, Green SC (2022). How variability shapes learning and generalization. Trends Cogn Sci..

[2] Battig WF, Bilodeau EA (1966). Facilitation and interference. Acquis Ski.

[3] Lin CHJ, Knowlton BJ, Chiang MC, Iacoboni M, Udompholkul P, Wu AD (2011). Brain–behavior correlates of optimizing learning through interleaved practice. Neuroimage..

[4] Schorn JM, Knowlton BJ (2021). Interleaved practice benefits implicit sequence learning and transfer. Mem. Cogn..

[5] Kim, T., Wright, D.L. & Feng, W. Commentary: Variability of practice, information processing, and decision making—How much do we know? *Front. Psychol*. [*Internet*]. 10.3389/fpsyg.2021.685749/full. Accessed 12 Aug 2021 (2021). 10.3389/fpsyg.2021.685749 PMC8371322 34421736

[6] Shea, J.B. & Morgan, R.L. Contextual interference effects on the acquisition, retention, and transfer of a motor skill. *J. Exp. Psychol. Hum. Learn. Mem*. [*Internet*] **5**, 179–187. http://content.apa.org/journals/xlm/5/2/179. Accessed 29 Sep 2017 (1979).

[7] Brady, F. Contextual interference: A meta-analytic study. *Percept. Mot. Skills* [*Internet*]. **99**, 116–126. 10.2466/pms.99.1.116-126 (2017). 10.2466/pms.99.1.116-126 15446636

[8] Coker CA (2017). Motor Learning and Control for Practitioners.

[9] Magill RA, Anderson DI (2017). Motor Learning and Control: Concepts and Applications.

[10] Wright DL, Kim T, Hodges NJ, Williams AM (2020). Contextual interference: New findings, insights, and implications for skill acquisition. Skill Acquisition in Sport: Research, Theory and Practice.

[11] Al-Mustafa, A.A. *Contextual Interference: Laboratory Artifact or Sport Skill Learning Related. Unpublished Dissertation*. (University of Pittsburgh, 1989).

[12] Barreiros, J., Figueiredo, T. & Godinho, M. The contextual interference effect in applied settings. *Eur. Phys. Educ. Rev*. [*Internet*] **13**, 195–208 10.1177/1356336X07076876 (2007).

[13] Lee TD, Simon D, Williams AM, Hodges EJ (2004). Contextual interference. Skill Acquisition in Sport: Research, Theory and Practice.

[14] Magill, R.A. & Hall, K.G. A review of the contextual interference effect in motor skill acquisition. *Hum. Mov. Sci*. [*Internet*] **9**, 241–89. http://www.sciencedirect.com/science/article/pii/016794579090005X (1990).

[15] Merbah, S. & Meulemans, T. Learning a motor skill: Effects of blocked versus random practice a review. *Psychol. Belg*. [*Internet*] **51**, 15–48. http://orbi.ulg.ac.be/bitstream/2268/105261/1/Merbah&Meulemans2011PsychologicaBelgica.pdf (2011).

[16] Wright, D., Verwey, W., Buchanen, J., Chen, J., Rhee, J. & Immink, M. Consolidating behavioral and neurophysiologic findings to explain the influence of contextual interference during motor sequence learning. *Psychon. Bull. Rev*. [*Internet*] **23**, 1–21 10.3758/s13423-015-0887-3. Accessed 25 Sep 2017 (2016). 10.3758/s13423-015-0887-3 26084879

[17] Henz, D., John, A., Merz, C. & Schöllhorn, W.I. Post-task effects on EEG brain activity differ for various differential learning and contextual interference protocols. *Front. Hum. Neurosci*. (2018). 10.3389/fnhum.2018.00019 PMC5797795 29445334

[18] Ammar A, Trabelsi K, Boujelbane MA, Boukhris O, Glenn JM, Chtourou H (2023). The myth of contextual interference learning benefit in sports practice: A systematic review and meta-analysis. Educ. Res. Rev..

[19] Brady, F. A theoretical and empirical review of the contextual interference effect and the learning of motor skills. *Quest* [*Internet*] **50**, 266–293. 10.1080/00336297.1998.10484285. Accessed 26 Sep 2017 (2017).

[20] Lee TD, Hodges NJ, Williams AM (2012). Contextual interference: Generalizability and limitations. Skill Acquisition in Sport: Research, Theory and Practice.

[21] Lage GM, Faria LO, Ambrósio NFA, Borges AMP, Apolinário-Souza T (2021). What is the level of contextual interference in serial practice? A meta-analytic review. J. Mot. Learn. Dev..

[22] Graser, J. V., Bastiaenen, C.H.G. & van Hedel, H.J.A. The role of the practice order: A systematic review about contextual interference in children. *PLoS One* **14** (2019). 10.1371/journal.pone.0209979 PMC6342307 30668587

[23] Sattelmayer, M., Elsig, S., Hilfiker, R. & Baer, G. A systematic review and meta-analysis of selected motor learning principles in physiotherapy and medical education. *BMC Med. Educ*. (BioMed Central Ltd.) (2016). 10.1186/s12909-016-0538-z PMC4714441 26768734

[24] Methley, A.M., Campbell, S., Chew-Graham, C., McNally, R. & Cheraghi-Sohi, S. PICO, PICOS and SPIDER: A comparison study of specificity and sensitivity in three search tools for qualitative systematic reviews. *BMC Health Serv. Res.* (2014). 10.1186/s12913-014-0579-0 PMC4310146 25413154

[25] Page MJ, Moher D (2017). Evaluations of the uptake and impact of the Preferred Reporting Items for Systematic reviews and Meta-Analyses (PRISMA) statement and extensions: A scoping review. Syst. Rev..

[26] Thomas H, Ciliska D, Dobbins M, Micucci S (2004). A process for systematically reviewing the literature: Providing the research evidence for public health nursing interventions. Worldviews Evid.-Based Nurs..

[27] Diekelmann S, Born J (2010). The memory function of sleep. Nat. Rev. Neurosci..

[28] Yang G, Lai CSW, Cichon J, Ma L, Li W, Gan WB (2014). Sleep promotes branch-specific formation of dendritic spines after learning. Science (80-).

[29] Dundar, Y. & Fleeman, N. Applying inclusion and exclusion criteria. In *Doing a Systematic Review: A Student's Guide* (Boland, A., Cherry, G.M., Dickson, R. eds.). 79–91 (SAGE, 2017).

[30] Shi J, Luo D, Weng H, Zeng X-T, Lin L, Chu H (2020). Optimally estimating the sample standard deviation from the five-number summary. Res. Synth. Methods.

[31] Shi, J., Luo, D., Wan, X., Liu, Y., Liu, J., Bian, Z. *et al*. Detecting the skewness of data from the sample size and the five-number summary. *ArXiv* (2020). 10.1177/09622802231172043 37161735

[32] Wan X, Wang W, Liu J, Tong T (2014). Estimating the sample mean and standard deviation from the sample size, median, range and/or interquartile range. BMC Med. Res. Methodol..

[33] Luo D, Wan X, Liu J, Tong T (2018). Optimally estimating the sample mean from the sample size, median, mid-range, and/or mid-quartile range. Stat. Methods Med. Res..

[34] Deeks, J. & Higgins, J. Statistical algorithms in review manager 5. *Stat. Methods Gr. Cochrane Collab*. [*Internet*]. https://training.cochrane.org/handbook/current/statistical-methods-revman5 (2010).

[35] Higgins, J.P.T. & Deeks, J. Chapter 6: Choosing effect measures and computing estimates of effect. In (Higgins, J., Thomas, J., Chandler, J., Cumpston, M., Li, T., Page, M, *et al*. eds.) *Cochrane Handbook for Systematic Reviews of Interventions* Version 63 [Internet]. https://www.training.cochrane.org/handbook (Cochrane, 2022).

[36] Higgins, J.P.T., Thomas, J., Chandler, J., Cumpston, M., Li, T., Page, M.J. *et al*. *Cochrane Handbook for Systematic Reviews of Interventions | Cochrane Trainin*g. Version 6.2 (updated Feb 2021). (Cochrane, 2021).

[37] Deeks JJ, Higgins JPT, Altman DG (2019). Analysing data and undertaking meta-analyses. Cochrane Handb Syst Rev Interv..

[38] Cohen, J. *Statistical Power Analysis for the Behavioral Sciences*. 2nd Ed. (Routledge, 1988).

[39] Assink M, Wibbelink CJM (2016). Fitting three-level meta-analytic models in R: A step-by-step tutorial. Quant. Methods Psychol..

[40] Cheung MWL (2014). Modeling dependent effect sizes with three-level meta-analyses: A structural equation modeling approach. Psychol. Methods.

[41] Van den Noortgate, W., López-López, J.A., Marín-Martínez, F. & Sánchez-Meca, J. Three-level meta-analysis of dependent effect sizes. *Behav. Res. Methods* [*Internet*] **45**, 576–594 10.3758/s13428-012-0261-6. Accessed 28 Apr 2024 (2013). 10.3758/s13428-012-0261-6 23055166

[42] Becker BJ, Tinsley HEA, Brown ED (2000). Multivariate meta-analysis. The Handbook of Applied Multivariate Statistics and Mathematical Modeling.

[43] Cheung SF, Chan DKS (2008). Dependent correlations in meta-analysis. Educ. Psychol. Meas..

[44] Thomas H (2003). Quality Assessment Tool for Quantitative Studies. Effective Public Health Practice Project.

[45] Moher D, Shamseer L, Clarke M, Ghersi D, Liberati A, Petticrew M (2016). Preferred reporting items for systematic review and meta-analysis protocols (PRISMA-P) 2015 statement. Rev. Esp. Nutr. Hum. Diet..

[46] Ste-Marie DM, Clark SE, Findlay LC, Latimer AE (2010). High levels of contextual interference enhance handwriting skill acquisition. J. Mot. Behav..

[47] Porter JM, Magill RA (2010). Systematically increasing contextual interference is beneficial for learning sport skills. J. Sport Sci..

[48] Shea CH, Lai Q, Wright DL, Immink M, Black C (2001). Consistent and variable practice conditions: Effects on relative and absolute timing. J. Mot. Behav..

[49] Chua LK, Dimapilis MK, Iwatsuki T, Abdollahipour R, Lewthwaite R, Wulf G (2019). Practice variability promotes an external focus of attention and enhances motor skill learning. Hum. Mov. Sci..

[50] Broadbent, D.P., Causer, J., Williams, A.M. & Ford, P.R. The role of error processing in the contextual interference effect during the training of perceptual-cognitive skills. *J. Exp. Psychol. Hum. Percept. Perform*. [*Internet*] **43**, 1329–1342 /fulltext/2017-12046-001.html (2017). 10.1037/xhp0000375 28301186

[51] Sherwood DE (1996). The benefits of random variable practice for spatial accuracy and error detection in a rapid aiming task. Res. Q. Exerc. Sport..

[52] Beik M, Taheri H, Saberi Kakhki A, Ghoshuni M (2020). Neural mechanisms of the contextual interference effect and parameter similarity on motor learning in older adults: An EEG study. Front. Aging Neurosci..

[53] Wong AWK, Whitehill TL, Ma EPM, Masters R (2013). Effects of practice schedules on speech motor learning. Int. J. Speech Lang. Pathol..

[54] Porter, J.M., Landin, D., Hebert, E.P. & Baum, B. The effects of three levels of contextual interference on performance outcomes and movement patterns in golf skills. *Int. J. Sports Sci. Coach*. **2**, 243–255 10.1260/174795407782233100 (2007).

[55] Porter JM, Saemi E (2010). Moderately skilled learners benefit by practicing with systematic increases in contextual interference. Int. J. Coach Sci..

[56] Del Rey P, Liu X, Simpson KJ (1994). Does retroactive inhibition influence contextual interference effects?. Res. Q. Exerc. Sport.

[57] French KE, Rink JE, Werner PH (1990). Effects of contextual interference on retention of three volleyball skills. Percept. Mot. Skills.

[58] Goodwin JE, Meeuwsen HJ (1996). Investigation of the contextual interference effect in the manipulation of the motor parameter of over-all force. Percept. Mot. Skills.

[59] Naimo MA, Zourdos MC, Wilson JM, Kim JS, Ward EG, Eccles DW (2013). Contextual interference effects on the acquisition of skill and strength of the bench press. Hum. Mov. Sci..

[60] Beik M, Taheri H, Saberi Kakhki A, Ghoshuni M (2021). Algorithm-based practice schedule and task similarity enhance motor learning in older adults. J. Mot. Behav..

[61] Kim T, Kim H, Wright DL (2021). Improving consolidation by applying anodal transcranial direct current stimulation at primary motor cortex during repetitive practice. Neurobiol. Learn. Mem..

[62] Porter C, Greenwood D, Panchuk D, Pepping GJ (2020). Learner-adapted practice promotes skill transfer in unskilled adults learning the basketball set shot. Eur. J. Sport Sci..

[63] Kaipa R, Mariam KR (2018). Role of constant, random and blocked practice in an electromyography-based oral motor learning task. J. Mot. Behav..

[64] Parab S, Bose M, Ganesan S (2018). Influence of random and blocked practice schedules on motor learning in children aged 6–12 years. Crit. Rev. Phys. Rehabil. Med..

[65] Li Y, Lima RP (2002). Rehearsal of task variations and contextual interference effect in a field setting. Percept. Mot. Skills.

[66] Green S, Sherwood DE (2000). The benefits of random variable practice for accuracy and temporal error detection in a rapid aiming task. Res. Q. Exerc. Sport.

[67] Brady F (1997). Contextual interference and teaching golf skills. Percept. Mot. Skills.

[68] Fazeli D, Taheri HR, Saberi KA (2017). Random versus blocked practice to enhance mental representation in golf putting. Percept. Mot. Skills.

[69] Bortoli L, Robazza C, Durigon V, Carra C (1992). Effects of contextual interference on learning technical sports skills. Percept. Mot. Skills.

[70] Pasand F, Fooladiyanzadeh H, Nazemzadegan G (2016). The effect of gradual increase in contextual interference on acquisition, retention and transfer of volleyball skillsce on acquisition, retention and transfer of volleyball skills. Int. J. Kinesiol. Sport Sci..

[71] Zetou E, Michalopoulou M, Giazitzi K, Kioumourtzoglou E (2007). Contextual interference effects in learning volleyball skills. Percept Mot. Skills.

[72] Cheong JPG, Lay B, Robert Grove J, Medic N, Razman R (2012). Practicing field hockey skills along the contextual interference continuum: A comparison of five practice schedules. J. Sports Sci. Med..

[73] Cheong JPG, Lay B, Razman R (2016). Investigating the contextual interference effect using combination sports skills in open and closed skill environments. J. Sports Sci. Med..

[74] Jeon MJ, Jeon HS, Yi CH, Kwon OY, You SH, Park JH (2020). Block and random practice: A wii fit dynamic balance training in older adults. Res. Q Exerc. Sport.

[75] Broadbent DP, Causer J, Ford PR, Williams AM (2015). Contextual interference effect on perceptual-cognitive skills training. Med. Sci. Sports Exerc..

[76] Frömer R, Stürmer B, Sommer W (2016). (Don’t) Mind the effort: Effects of contextual interference on ERP indicators of motor preparation. Psychophysiology..

[77] Moreno J, Avila F, Damas S, Luis V, Reina L (2003). Contextual interference in learning precision skills. Percept. Mot. Skills.

[78] Bertollo M, Berchicci M, Carraro A, Comani S, Robazza C (2010). Blocked and random practice organization in the learning of rhythmic dance step sequences. Percept. Mot. Skills.

[79] Pollatou E, Kioumourtzoglou E, Agelousis N, Mavromatis G (1997). Contextual interference effects in learning novel motor skills. Percept. Mot. Skills.

[80] Jiménez-Díaz J, Morera-Castro M, Salazar W (2018). The contextual interference effect on the performance of fundamental motor skills in adults. Hum. Mov..

[81] Saemi E, Porter JM, Ghotbi Varzaneh A, Zarghami M, Shafinia P (2012). Practicing along the contextual interference continuum: A comparison of three practice schedules in an elementary physical education setting. Kinesiology..

[82] Tsutsui S, Satoh M, Yamamoto K (2013). Contextual interference modulated by pitcher skill level. Int. J. Sport Health Sci..

[83] Vera JG, Montilla MM (2003). Practice schedule and acquisition, retention, and transfer of a throwing task in 6-yr-old children. Percept. Mot. Skills.

[84] Smith PJK, Davies M (1995). Applying contextual interference to the Pawlata roll. J. Sports Sci..

[85] Moretto NA, Marcori AJ, Okazaki VHA (2018). Contextual interference effects on motor skill acquisition, retention and transfer in sport riffle schooting. Hum. Mov..

[86] Rivard JD, Vergis AS, Unger BJ, Gillman LM, Hardy KM, Park J (2015). The effect of blocked versus random task practice schedules on the acquisition and retention of surgical skills. Am. J. Surg..

[87] Shewokis PA, Shariff FU, Liu Y, Ayaz H, Castellanos A, Lind DS (2017). Acquisition, retention and transfer of simulated laparoscopic tasks using fNIR and a contextual interference paradigm. Am. J. Surg..

[88] Li Y, Wright DL (2000). An assessment of the attention demands during random- and blocked-practice schedules. Q. J. Exp. Psychol. Sect. A Hum. Exp. Psychol..

[89] Simon DA (2007). Contextual interference effects with two tasks. Percept. Mot. Skills.

[90] Simon DA, Lee TD, Cullen JD (2008). Win-shift, lose-stay: Contingent switching and contextual interference in motor learning. Percept. Mot. Skills.

[91] Wright DL, Li Y, Whitacre C (1992). The contribution of elaborative processing to the contextual interference effect. Res. Q. Exerc. Sport.

[92] Lin CH, Yang HC, Knowlton BJ, Wu AD, Iacoboni M, Ye YL (2018). Contextual interference enhances motor learning through increased resting brain connectivity during memory consolidation. Neuroimage..

[93] Lin CHJ, Chiang MC, Wu AD, Iacoboni M, Udompholkul P, Yazdanshenas O (2012). Age related differences in the neural substrates of motor sequence learning after interleaved and repetitive practice. Neuroimage..

[94] Beik M, Fazeli D (2021). The effect of learner-adapted practice schedule and task similarity on motivation and motor learning in older adults. Psychol. Sport Exerc..

[95] Chalavi S, Pauwels L, Heise KF, Zivariadab H, Maes C, Puts NAJ (2018). The neurochemical basis of the contextual interference effect. Neurobiol. Aging.

[96] Immink MA, Pointon M, Wright DL, Marino FE (2021). Prefrontal cortex activation during motor sequence learning under interleaved and repetitive practice: A two-channel near-infrared spectroscopy study. Front. Hum. Neurosci..

[97] Russell DM, Newell KM (2007). How persistent and general is the contextual interference effect?. Res. Q. Exerc. Sport.

[98] Porter JM, Beckerman T (2016). Practicing with gradual increases in contextual interference enhances visuomotor learning. Kinesiology..

[99] Smith PJK (2002). Task duration in contextual interference. Percept. Mot. Skills.

[100] Kim T, Chen J, Verwey WB, Wright DL (2018). Improving novel motor learning through prior high contextual interference training. Acta Psychol. Amst..

[101] Thomas JL, Fawver B, Taylor S, Miller MW, Williams AM, Lohse KR (2021). Using error-estimation to probe the psychological processes underlying contextual interference effects. Hum. Mov. Sci..

[102] Broadbent DP, Causer J, Mark Williams A, Ford PR (2017). The role of error processing in the contextual interference effect during the training of perceptual-cognitive skills. J. Exp. Psychol. Hum. Percept. Perform..

[103] Aiken CA, Genter AM (2018). The effects of blocked and random practice on the learning of three variations of the golf chip shot. Int. J. Perform. Anal. Sport.

[104] Waqqash E, Low J (2015). Effects of contextual interference (CI) in basic squash shots practice. Malays. J. Sport Sci. Recreat..

[105] Smith PJK, Davies M (2008). Applying contextual interference to the Pawlata roll. J. Sport Sci..

[106] De Souza MGTX, Nunes MES, Corrêa UC, Dos Santos S (2015). The contextual interference effect on sport-specific motor learning in older adults. Hum. Mov..

[107] Broadbent, D.P., Causer, J., Ford, P.R. & Williams, A.M. Contextual interference effect on perceptual-cognitive skills training. *Med. Sci. Sports Exerc*. [*Internet*] **47**, 1243–1250 https://pubmed.ncbi.nlm.nih.gov/25255127/ (2015). 10.1249/MSS.0000000000000530 25255127

[108] Van Aert RCM, Wicherts JM, Van Assen MALM (2019). Publication bias examined in meta-analyses from psychology and medicine: A meta-meta-analysis. PLoS One..

[109] Lee TD, White MA (1990). Influence of an unskilled model’s practice schedule on observational motor learning. Hum. Mov. Sci..

[110] Hebert EP, Landin D, Solmon MA (1996). Practice schedule effects on the performance and learning of low- and high-skilled students: An applied study. Res. Q. Exerc. Sport.

[111] Wulf G, Shea CH (2002). Principles derived from the study of simple skills do not generalize to complex skill learning. Psychon. Bull. Rev. Psychon. Soc. Inc..

[112] Landin D, Hebert EP (1997). A comparison of three practice schedules along the contextual interference continuum. Res. Q. Exerc. Sport.

[113] Sekiya H, Magill RA (2000). The contextual interference effect in learning force and timing parameters of the same generalized program. J. Hum. Mov. Stud..

[114] Sekiya H, Magill RA, Anderson DI (1996). The contextual interference effect in parameter modifications of the same generalized motor program. Res. Q. Exerc. Sport.

[115] Sekiya H, Magill RA, Sidaway B, Anderson DI (1994). The contextual interference effect for skill variations from the same and different generalized motor programs. Res. Q. Exerc. Sport.

[116] Wulf G, Lee TD (1993). Contextual interference in movements of the same class: Differential effects on program and parameter learning. J. Mot. Behav..

[117] Smith PJK (2002). Applying contextual interference to snowboarding skills. Percept. Mot. Skills.

[118] Smith PJK, Gregory SK, Davies M (2003). Alternating versus blocked practice in learning a cartwheel. Percept. Mot. Skills.

[119] Wrisberg CA, Liu Z (1991). The effect of contextual variety on the practice, retention, and transfer of an applied motor skill. Res. Q. Exerc. Sport..

[120] Hall KG, Magill RA (1995). Variability of practice and contextual interference in motor skill learning. J. Mot. Behav..

[121] Lee TD, Wulf G, Schmidt RA (1992). Contextual interference in motor learning: Dissociated effects due to the nature of task variations. Q. J. Exp. Psychol. Sect. A.

[122] Shea JB, Titzer RC (1993). The influence of reminder trials on contextual interference effects. J. Mot. Behav..

[123] Bortoli L, Spagolla G, Robazza C (2001). Variability effects on retention of a motor skill in elementary school children. Percept. Mot. Skills.

[124] Huedo-Medina T, Sanchez-Meca J, Marin-Martinez F, Botella J (2006). Assessing heterogeneity in meta-analysis: Q statistic or I^2^ index?. Psychol. Methods.

[125] Rücker G, Schwarzer G, Carpenter JR, Schumacher M (2008). Undue reliance on I^2^ in assessing heterogeneity may mislead. BMC Med. Res. Methodol..

[126] Schroll JB, Moustgaard R, Gøtzsche PC (2011). Dealing with substantial heterogeneity in Cochrane reviews. Cross-sectional study. BMC Med. Res. Methodol..

[127] Alba AC, Alexander PE, Chang J, Macisaac J, Defry S, Guyatt GH (2016). High statistical heterogeneity is more frequent in meta-analysis of continuous than binary outcomes. J. Clin. Epidemiol..

